# Functional Ionic Liquids Decorated Carbon Hybrid Nanomaterials for the Electrochemical Biosensors

**DOI:** 10.3390/bios11110414

**Published:** 2021-10-23

**Authors:** Pushpesh Ranjan, Shalu Yadav, Mohd Abubakar Sadique, Raju Khan, Jamana Prasad Chaurasia, Avanish Kumar Srivastava

**Affiliations:** 1 CSIR—Advanced Materials and Processes Research Institute (AMPRI), Hoshangabad Road, Bhopal 462026, India; pushpeshranjanmail@gmail.com (P.R.); shaluyadav1510@gmail.com (S.Y.); mabu1221@gmail.com (M.A.S.); jpchourasia@ampri.res.in (J.P.C.); avanish.aks555@gmail.com (A.K.S.); 2 Academy of Scientific and Innovative Research (AcSIR), Ghaziabad 201002, India

**Keywords:** ionic liquids, carbon nanomaterials, graphene, graphene oxide, electrochemical sensor, biosensors

## Abstract

Ionic liquids are gaining high attention due to their extremely unique physiochemical properties and are being utilized in numerous applications in the field of electrochemistry and bio-nanotechnology. The excellent ionic conductivity and the wide electrochemical window open a new avenue in the construction of electrochemical devices. On the other hand, carbon nanomaterials, such as graphene (GR), graphene oxide (GO), carbon dots (CDs), and carbon nanotubes (CNTs), are highly utilized in electrochemical applications. Since they have a large surface area, high conductivity, stability, and functionality, they are promising in biosensor applications. Nevertheless, the combination of ionic liquids (ILs) and carbon nanomaterials (CNMs) results in the functional ILs-CNMs hybrid nanocomposites with considerably improved surface chemistry and electrochemical properties. Moreover, the high functionality and biocompatibility of ILs favor the high loading of biomolecules on the electrode surface. They extremely enhance the sensitivity of the biosensor that reaches the ability of ultra-low detection limit. This review aims to provide the studies of the synthesis, properties, and bonding of functional ILs-CNMs. Further, their electrochemical sensors and biosensor applications for the detection of numerous analytes are also discussed.

## 1. Introduction

Ionic liquids (ILs) is a class of organic salt composed of organic cations containing heteroatoms, like nitrogen or phosphorus, and organic or inorganic anions, which exist in a liquid state below 100 °C. Numerous combinations of cationic ions, like tetraalkylammonium, tetra alkyl phosphonium, trialkyl sulfonium, imidazolium, pyridinium, pyrrolidinium, piperidinium, etc., and anionic halide ions, tetrafluoroborate, hexafluorophosphate, bis(trifluoromethyl sulfonyl)amide, dicyanamide, thiocyanate, and trifluoromethane-sulfonate, triflate, etc., are possible in ILs. ILs possess excellent ionic mobility, thermal stability, catalytic properties, and biocompatibility. Moreover, the remarkable biological and eco-friendly nature, i.e., low-hazardous state, low toxicity, and biodegradability, position them as the better choice in green chemistry processes [1,2,3]. In addition, they have excellent properties, such as high conductivity, wide electrochemical window, high stability, low volatility, moderate viscosity, non-flammability, and low melting point [4]. However, suitable combinations of cationic and anionic species could tune their structural properties to improve their physical and chemical characteristics, like solvation property, melting point, viscosity, density, polarity, low-vapor pressure, hydrophilicity, hydrophobicity, and ionic conductivity [1,5]. Due to these enormous properties, they are widely applicable in sensors [6,7], biosensors [8], electro-catalyst [9], energy storage devices [10,11], solar cells [12], thin-film membranes [13,14], tissue engineering [15], drug delivery systems [16], therapeutics [17], wound healing [18], and antimicrobial and antiviral agents [19].

Carbon and its related materials are being utilized in the application of electrochemical devices from a very early period. These are mainly zero-dimensional (0-D), such as graphene quantum dots (GQDs), carbon quantum dots (CQDs), carbon nanodiamonds (CNDs), and fullerene [20,21]; one-dimensional (1-D), such as single-walled carbon nanotubes (SWCNTs), multi-walled carbon nanotubes (MWCNTs), and carbon nanofibers (CNFs) [22]; and two-dimensional (2-D), like graphene (GR), graphene oxide (GO) [23,24], reduced graphene oxide (RGO), and graphene nanoribbons (GNRs) [25,26]. Few materials, like GQDs, CQDs, and CNDs, possess excellent optical and considerable electrochemical properties. Moreover, GR, SWCNTs, and MWCNTs offer high conductivity, low resistance, reproducibility, ease of functionalization, modification, and cost effectiveness. In addition, they possess extremely remarkable electronic and mechanical properties [27]. On the other hand, GO has lower conductivity than RGO. However, they possess high water dispersibility and are easy to modify. These remarkable properties open a new pathway that considerably allowed the use of the carbon nanomaterials (CNMs) for the construction of devices in biosensors applications. In this regard, various electrochemical biosensors have been developed for the detection of different kinds of biological and non-biological analytes. However, CNMs have limitations of robustness and long-term stability, and continuous research is being carried out to overcome these issues to enable their use in biosensing applications [28,29].

CDs are small-sized carbon nanomaterials having a diameter less than 10 nm. They are mainly comprised of GQDs, CQDs, and CNDs. They have excellent electro-optical and optical properties due to their quantum confinement and edge effects [30,31]. However, their considerable electrochemical properties attracted more consideration towards their applicability in the electrochemical biosensors since the synthesis strategies of the GQDs and CQDs are easy and cost-effective, and their size can be tuned according to the desired applications. Furthermore, the high oxygen functionality, water-solubility, large surface area, and heteroatom doping tendency increase their utility in numerous areas, such as biosensing and bioimaging. On the other hand, their low synthetic reproducibility, low conductivity, toxicity, and limited stability are still challenging in the case of CDs, which further restricted their applications [32,33]. Another derivative of carbon is nanodiamonds, where the synthesis of nanodiamonds is quite complex and is done at a high temperature, and somehow, the use of explosives in the detonation method may be dangerous. Nanodiamonds have superior optical properties, considerable hardness, chemical stability, high thermal conductivity, biocompatibility, very low toxicity, and sustainability in harsh conditions. However, their electro-conductivity is low but can be enhanced by the boron-doping, which could be used in the electrochemical analysis [34,35].

CNTs are considered as rolled GR sheets into a nanotube with a diameter of 0.4–100 nm. CNTs have magnificent physical and chemical properties and can be further tuned by their chemical modification. Moreover, the structural and compositional characteristics, such as orientation, arrangement, wall-thickness, length, and impurities of CNTs, affect their properties. Additionally, the high electro-conductivity, stability, and stiffness make them an ideal nanomaterial in electrochemical biosensor applications. However, some drawbacks of the CNTs are their expensive synthesis, difficulty to purify, and less control on the CNT structure, which limited their applications [36,37].

GR is an sp^2^-bonded carbon atom of a single-atom-thick sheet arranged in a honeycomb structure. It has extraordinary electrical conductivity, mechanical and optical properties, and a high surface-to-volume area (~2630 m^2^/g). Due to its excellent properties, it gained much attention in biosensor applications. However, the hydrophobic nature, low dispersibility, and lack of surface functionality limited its application in electrochemical biosensor-based diagnostics devices [38,39]. On the other hand, in the GR family, GO contains high oxygen functionality on its surface plane and edges. The oxygen functionality introduces the hydrophilic properties, which increase the dispersibility in the aqueous solution. However, the poor conductivity further restricted its application in biosensors [40]. Still, the reduction of GO through the chemical, thermal or electrochemical methods into RGO by reducing some magnitude of functionality results in the high conductive material as compared to GO. Therefore, the few defects that arise in the reduction process may contribute to the enhancement of the electrical property [41]. Non-linear optical responses of the GR, GO, and RGO directly governs their impedance, capacitance, photoconductivity, and photo-thermal behavior. It was found that the electrical capacitance of the RGO was diminished by the existence of oxygen functionalities. Furthermore, the capacitance of the materials is co-related with their electrical impedance as a function of their electrical frequency. Marin et al. reported that the materials exhibit capacitive response by reduction of the electrical impedance. However, the introduction of metallic nanoparticles (AuNPs) onto RGO enhances their capacitive response as compared to RGO [42].

**Figure 1:**
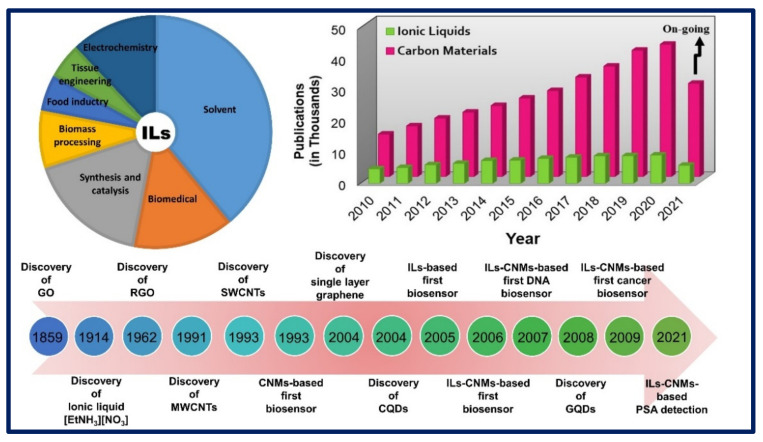
Pie chart of applications of ILs and historical discovery and applications perspective of ILs and carbon materials. Reprinted with permission from Ref. [3]. Copyright 2019, Royal Society of Chemistry; bar graph displays the number of publications per year from 2010 to 2021. (Source: Web of Science, Keyword: Ionic Liquids, and Carbon Materials, Last accessed date: 21 September 2021).

The biocompatibility, ease of modification, and synthesis of high-conductive nanocomposites of GO and RGO as well as simple electrode fabrication are the advantages of the GO and RGO in biosensor applications [43]. Figure 1 shows the discovery and applications of CNMs and ILs and their per-year publications, which display that ILs and CNMs are highly researchable areas nowadays [3,44,45,46,47,48,49,50,51].

Concerning the efficiency of electrochemical devices, the ionic conductivity of the materials and solvent media play an important role in enhancing the performance of these devices [52]. It can be observed that the electrolyte possesses high electron mobility and low viscosity, which could result in a high electrochemical stability frame. These enormous properties of ILs make them favorable in biosensor applications, mostly in the fabrication of electrochemical and optical sensors and biosensor devices. In addition, they serve as an electrolyte in numerous electrochemical reactions media. Owing to low volatility and a wide temperature range, they have great potential to replace the highly volatile, flammable, and hazardous solvents. Besides, the high viscosity and adhesion property of ILs introduces them as a good binder and enhances the immobilization of biomolecules on the sensor surfaces. Therefore, the sensitivity of the biosensor can be intensely enhanced and reach the ultra-low limit of detection (LOD) [8,53]. 

**Figure 2:**
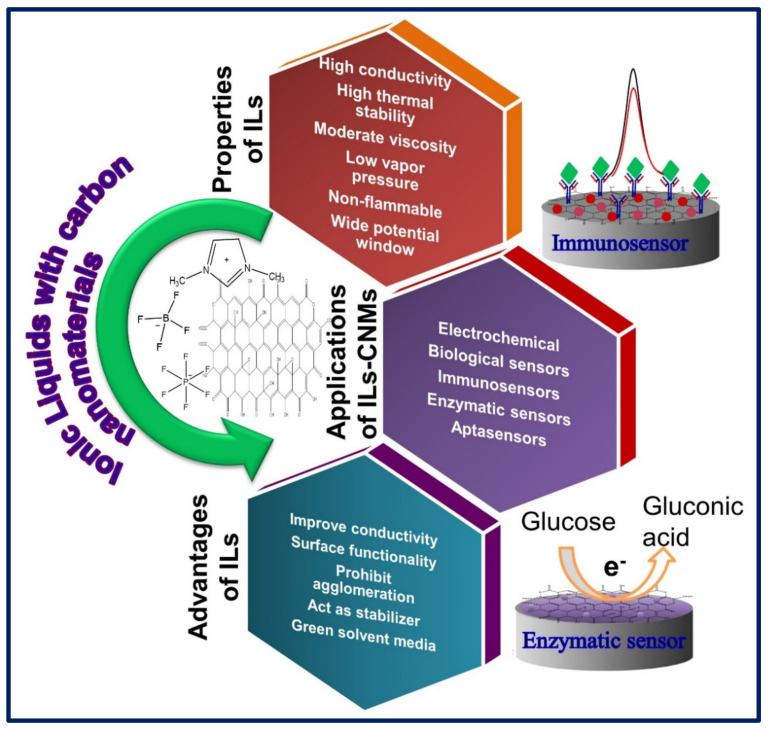
Representation of the properties, applications, and advantages of ILs-CNMs.

To date, an enormous number of electrochemical sensors and biosensors are reported based on CNMs that have superior performance in terms of sensitivity, stability, reusability, and reproducibility. However, the functionalization or modification with metal nanoparticles, enzymes, polymers, and heteroatom doping can improve their physiochemical properties and offers a great platform for diverse electrochemical biosensor methods [54,55]. On the other hand, the integration of CNMs with the functional ILs can turn to high-performance, hybrid nanostructured materials with superior properties even compared to the pure ILs and CNMs. These hybrid ILs-CNMs can significantly enhance the sensitivity, stability, and reproducibility of the sensor [56]. Figure 2 represents the properties, advantages, of ILs and applications of ILs-CNMs employed in biosensor applications.

In a review reported by Abo-Hamad and colleagues (in the year 2017), they compiled the information of the ILs-CNMs-based electrochemical sensor, their synthetic strategies and numerous sensing applications for biomarkers, metal ions, pharmaceutical drugs, and other chemicals. They mainly focused on the synthetic methods of ILs-CNMs, designing of sensors, and modifications [57]. Similarly, Verma and Ebenso (in the year 2018) gathered the information of the IL’s functionalized graphene materials, their functionalization, bonding, and numerous applications, including pollutants detection, hydrogen generation, CO_2_ capture, and catalysis application. However, this review does not specifically describe the sensors and biosensor application [58]. These reviews systematically presented the synthesis and diverse applications of ILs-CNMs, but they lacked information regarding biosensor applications for the detection of several cancerous and cardiac biomarkers as well as other analytes whose functions are directly correlated to the biological processes. Furthermore, these reviews are not updated with the recent work in the field of ILs-CNMs.

In this review, we will discuss the synthetic strategies, physical and chemical properties, and bonding involved in the ILs-CNMs. Furthermore, various ILs-functionalized hybrid carbon nanostructured-based fabrications of electrochemical sensors and biosensors, their working principle, advantages, and potential application are also discussed. This review would be helpful and will provide a good platform for academicians, researchers, and others to design and develop ILs-CNMs-based high-performance, advanced electrochemical biosensors for diagnostic applications.

## 2. Synthesis of Functional Ionic Liquid-Carbon Hybrid Nanomaterials.

There have been several methods, such as direct mixing [59], ultrasonication [60], hydrothermal [61], electrodeposition [62], microwave irradiation [63], layer-by-layer assembly [64], and sol-gel [65], reported for the preparation of ILs functionalized hybrid CNMs. Over the several methods, microwave irradiation, ultrasound-assisted, hydrothermal and direct mixing garnered high attention due to their several advantages, like easy, fast, and task-specific, high-yield, and environmentally friendly synthesis [66,67].

**Figure 3:**
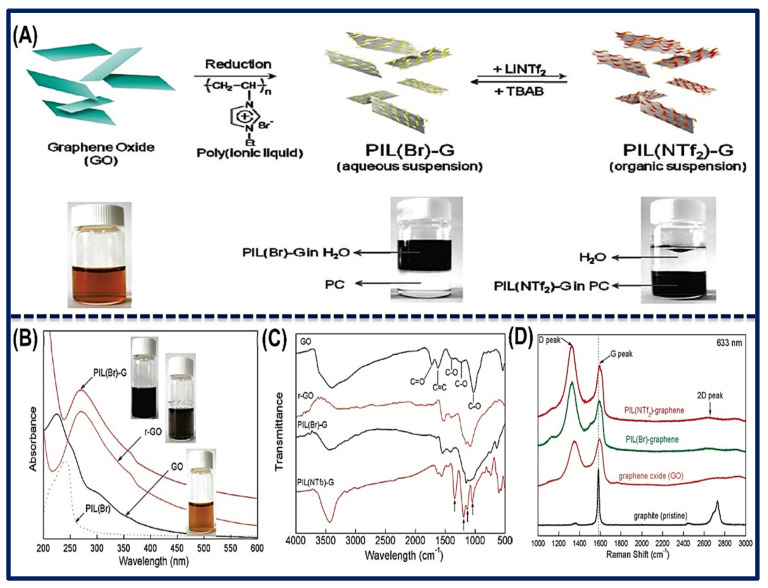
(**A**) Schematic illustration of the synthetic process of the (PIL)-G sheet and anion exchange of PIL, leading to the phase transfer of PIL-modified GR sheets between aqueous and organic solvent media. (**B**) UV-vis spectra and (**C**) FT-IR spectra of GO, RGO, an aqueous suspension of PIL(Br)-G, and an organic phase suspension of PIL(NTf_2_^−^)-G. (**D**) Raman spectra of pristine graphite, GO, PIL(Br)-G, and PIL(NTf_2_^−^)-G. Reprinted with permission from Ref. [68]. Copyright 2010, American Chemical Society.

Kim et al. synthesized the polyionic liquid (PIL)-functionalized RGO sheet in an aqueous and organic solvent medium. Herein, an aqueous solution of poly (1-vinyl-3-ethylimidazolium) bromide (PIL) was added into GO suspension and allowed to react and then reduced using hydrazine hydrate to form PIL (Br)-G sheet, where conductivity of the PIL (Br)-G was calculated to be ~36.0 S/cm. Further lithium bis(trifluoromethylsulfonyl) amide (Li^+^NTf_2_^−^) was added into the synthesized composites to allow the reversible phase transfer process, where the exchange of anion of the ionic-liquids-functionalized with the GR sheet takes place. As a result, it converted to PIL (NTf_2_^−^)-G, which inverted their properties from hydrophilic to hydrophobic. However, the PIL(Br)-G could be further restored by the treatment of PIL(NTf_2_^−^)-G, with the bromide anion containing ILs like tetrabutylammonium bromide (TBAB) or tetra-butyl phosphonium bromide (TBPB). It was observed that the PIL of the GR solution prohibited their agglomeration, which stabilized the solution for up to six months. Figure 3A illustrates the synthetic process and anion exchange of the (PIL)-G and PIL (NTf_2_^−^)-G sheets between aqueous and organic solvent media. Furthermore, PIL (Br)-G was characterized through the UV-Vis spectroscopy, where the absorption maximum of GO was found to be 230 nm; however, for RGO and PIL (Br)-G, it was ~270 nm. The bathochromic shift was due to the restoration of π-conjugation in the reduced form of GO. In addition, the FTIR spectroscopy explored the functional groups (C=O, C-O, C=C, C-C, etc.) present in the nanomaterial and cooperative studies of the shifting of the peak. On the other hand, Raman analysis of pristine GR displays the G and 2D peaks at 1582 cm^−1^ and 2726 cm^−1^, respectively. However, the G peak in GO is broader, and D is a notable peak. Results suggested that graphite is oxidized into GO, causing reduction of the size of in-plane sp^2^ moieties. Furthermore, (PIL)-G sheet displays the G, D, and 2D peaks at 1591 cm^−1^, 1324 cm^−1^, and 2642 cm^−1^, respectively. Conferring the above result, it was observed that in (PIL)-G sheet, the number of layers decreases and is stabilized during the reduction of GO. Figure 3B–D shows the analysis of nanomaterials via UV-Vis, FTIR, and Raman spectroscopy [68].

**Figure 4:**
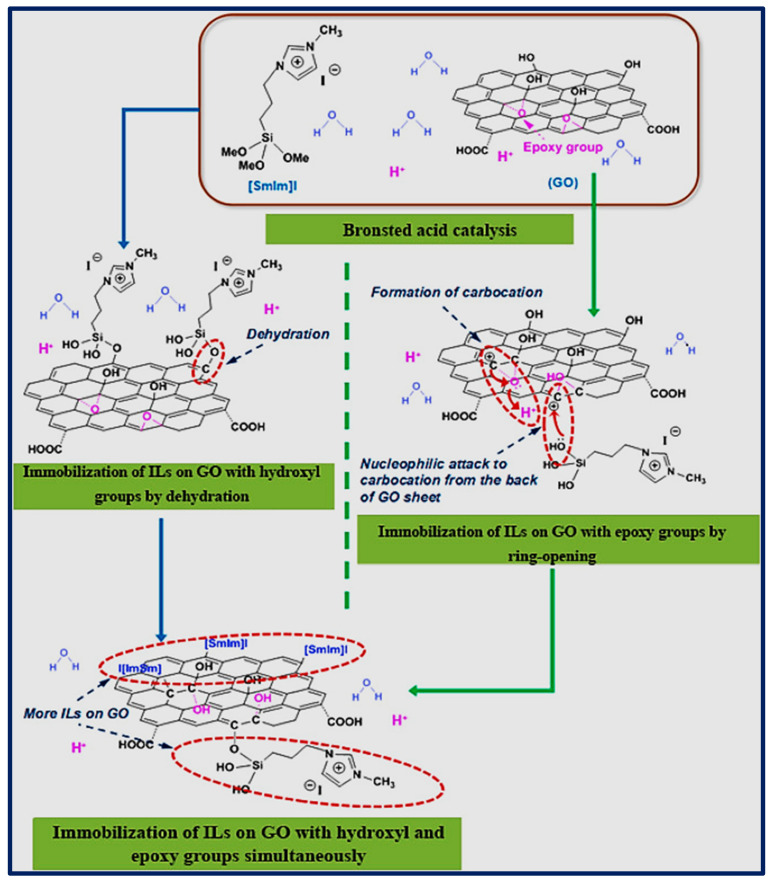
The mechanism for ILs immobilization on GO sheets in the acid medium. Reprinted with permission from Ref. [69]. Copyright 2018, Elsevier.

In a study by Zhu et al., they prepared the functional hybrid nanomaterials of GO-ILs using acid to catalyze the reaction. Therein, the imidazolium-based ILs containing the 1-(trimethoxysilyl) propyl-3-methylimidazolium halide cation and iodine or bromide anion and GO were added into aqueous acid solution (aq. HCl or aq. H_2_SO_4_) and sonicated to mix them, followed by heating at 50 °C for 12 h to allow the functionalization of ILs on GO surface. Further, several steps were done to purify the crude mixture. It was observed that in most cases, ILs can only bind to the hydroxyl group or through the ring-opening reaction by NH_2_ containing ILs. However, in this case, a one-step acid catalysis reaction favors the mechanism of ILs to immobilize on both hydroxyl and epoxy groups. Hence, the amount of deposition of ILs on GO can be significantly improved. The mechanism for ILs immobilization on GO sheets in an acidic medium is illustrated in Figure 4 [69]. 

**Figure 5:**
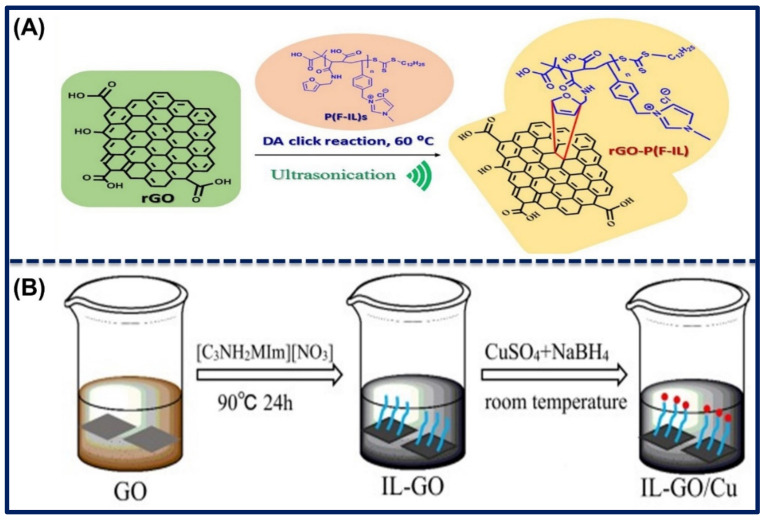
(**A**) Synthetic scheme for the preparation of RGO-Poly(F-IL) hybrids. Reprinted with permission from Ref. [70]. Copyright 2020, Elsevier; and (**B**) GO-ILs/CuNPs composite. Reprinted with permission from Ref. [71]. Copyright 2019, Elsevier.

**Figure 6:**
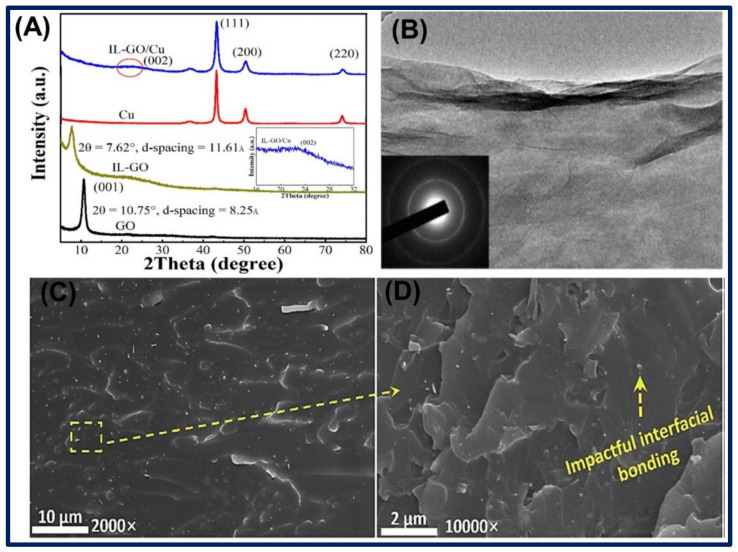
(**A**) XRD patterns of GO, GO-ILs, Cu, and GO-ILs/CuNPs. (**B**) TEM image of GO-ILs. Reprinted with permission from Ref. [71]. Copyright 2019, Elsevier. (**C**,**D**) SEM images of the fracture surface of EP/AIL-MWCNTs composites at 2.0 wt.% filler contents. Reprinted with permission from Ref. [72]. Copyright 2019, Elsevier.

In another study, ILs functionalized RGO nanocomposites were synthesized via Diels–Alder click reaction. They mixed the PIL in RGO solution and ultrasonicated it for few hours to thoroughly mix and functionalize ILs on the RGO surface. The crude materials were purified and heated until dried to obtain pure ILs-RGO nanomaterials. The illustration for the synthetic preparation of RGO-Poly(F-IL) hybrids material is shown in Figure 5A. The nanomaterials were characterized through the different characterization techniques, and one of the Raman analyses revealed that the ILs covalently bound onto the RGO surface, where some sp^2^ centers convert into sp^3^ carbon atoms. The authors employed the ILs-RGO nanocomposites in energy storage applications for the construction of supercapacitors [70]. Similarly, Gan et al. prepared amine-terminated ILs-GO nanocomposites through the ultra-sonication method in a basic solution. They mixed the 1-aminoethyl-3-methylimidazolium nitrate ILs with GO in KOH solution and further ultra-sonicated to disperse well, followed by the heating of the mixture at 90 °C for 24 h to form GO-ILs nanocomposites. Basic media improved the solubility of GO in solution as well as avoided the interaction of ILs with water. Therefore, resultant products were highly functionalized and stable nanocomposites. Further, the impurities of the nanocomposite solution were removed by several steps, including centrifugation, washing, and followed by the neutralization of the pH of the solution. In addition, they also synthesized the nanocomposites of GO-ILs-CuNPs by reduction of CuSO_4_ in GO-ILs solution by sodium borohydride at room temperature in a similar basic solution. A schematic of the preparation of GO-ILs and GO-ILs-CuNPs composite is shown in Figure 5B. XRD analyses were done to determine the interlayer change, phase structure, and particle size of the nanomaterials. GO and GO-ILs show the (001) diffraction peak at 2θ = 10.8° and 7.6°, with the interlayer spacing of 8.25 Ả and 11.61 Ả. The result suggested that ILs functionalized onto the GO via amide and ring-opening reaction. TEM image revealed that the GO-ILs have a well-ordered and high-quality structure with some wrinkles and ripples on the surface. Figure 6A,B shows the XRD patterns of GO, GO-ILs, Cu, and GO-ILs-CuNPs and a TEM image of GO-ILs [71]. 

**Figure 7:**
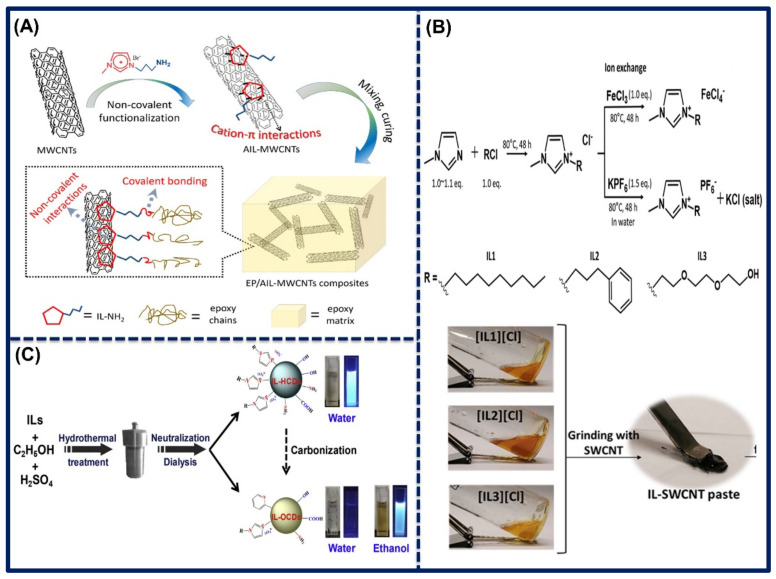
Synthesis of (**A**) EP/AIL-MWCNTs nanocomposites. Reprinted with permission from Ref. [72]. Copyright 2019, Elsevier. (**B**) Methyl imidazole-based ILs with Cl^−^ and anion exchanges from Cl^−^ to FeCl_4_^−^ and PF_6_^−^ for each ILs and ILs-SWCNT pastes. Reprinted with permission from Ref. [73]. Copyright 2018, American Chemical Society. (**C**) ILs-HCDs and ILs-OCDs. Reprinted with permission from Ref. [74]. Copyright 2017, Elsevier.

Chen et al. prepared the amine-functionalized ILs by refluxing the 3-Bromopropylamine hydrobromide and N-methylimidazole precursors in ethanol in a particular proportion. Further, the as-prepared ILs were mixed with the acid-functionalized MWCNTs, which are dispersed in dimethylformamide and stirred for 24 h at room temperature. However, 2-ethyl-4-methylimidazole (EMI-2,4) as a hardener was used to synthesize the epoxy functionalized ILs-MWCNTs nanocomposites. SEM image showed that after the reaction, and ILs functionalized on MWCNTs, there have been no morphological or structural changes, and no damages were noted. Therefore, this was the considerable method to synthesize the IL-MWCNTs nanocomposites. SEM images of the surface of EP/AIL-MWCNTs nanocomposites at 2.0 wt.% filler contents are shown in Figure 6C,D. The synthesis of EP/AIL-MWCNTs nanocomposites is illustrated in Figure 7A [72]. Park et al. synthesized the [NMIM][Cl], 1-(3-phenyl propyl)-3-methylimidazolium chloride, and 1-(2-(2-(2-hydroxyethoxy)ethoxy)ethyl)-3-methylimidazolium chloride ILs from their precursors and heated at 80 °C for 48 h and then purified in acetone, tetrahydrofuran, and ethyl ether to remove unreacted molecules and further dried in the vacuum oven. Afterward, synthesized ILs were mixed with SWCNTs in an agate mortar to prepare the ILs-SWCNT paste, which was implemented for the fabrication of a sensor to detect the volatile organic compounds in human breath. Synthesis of ILs and ILs-SWCNT pastes are shown in Figure 7B [73]. 

Hydrothermal method: Shu et al. synthesized the imidazolium-based IL functionalized CDs using different ILs. By the process, 1.0 g of specific IL was mixed with the ethanol and sulphuric acid, placed in a hydrothermal container, and heated at 200 °C for 48 h. Then, a brown-colored solution was obtained and purified, and the carbon dots (HCDs), which contain hydrophilic properties, were collected. Furthermore, organophilic carbon dots (OCDs) were obtained by the decanting of the HCDs. The yield of the ILs-functionalized HCDs and OCDs was found between 2.69–8.35% and 0–24.8%, respectively. Greenish-blue photoluminescence was shown by the above-synthesized ILs-CDs. In addition, OCDs exhibited high stability for up to six months without any aggregation. Schematic illustration for the preparation of IL-HCDs and IL-OCDs is shown in Figure 7C [74]. 

**Figure 8:**
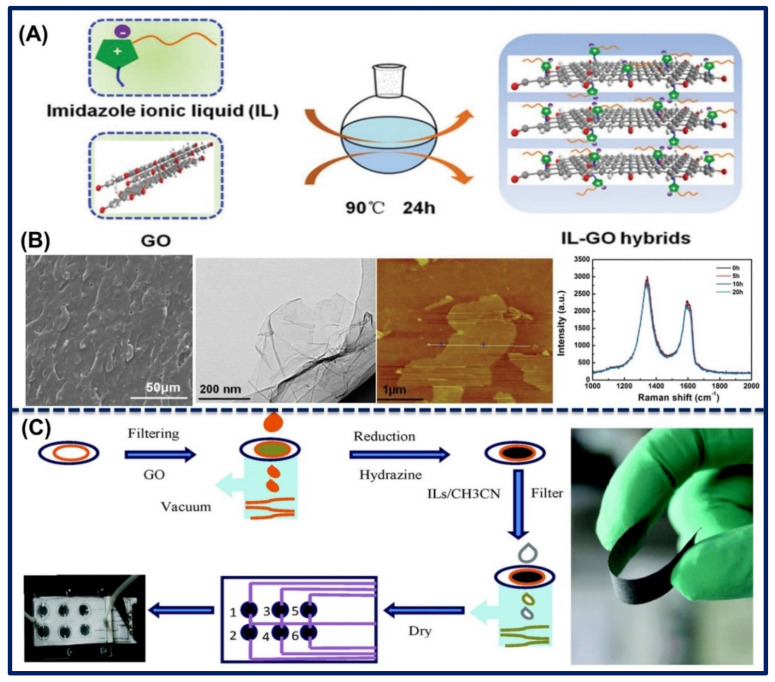
(**A**) Schematic of the preparation of GO-ILs hybrids nanocomposites. (**B**) SEM, TEM, and SPM images of GO-ILs hybrids and stability study of the GO-ILs hybrid aqueous solution using the Raman spectrum. Reprinted with permission from Ref. [76]. Copyright 2018, Royal Society of Chemistry. (**C**) The procedure for the preparation of flexible, paper-supported RGO-ILs sensor array. Reprinted with permission from Ref. [77]. Copyright 2016, Royal Society of Chemistry.

Direct mixing: In a synthetic approach, Zhou et al., synthesized the nanocomposites of four different combinations of ILs using (APMIM) and (AEMIM) cation and Br and (NTf_2_) anion with GO. For the proper binding, firstly, they activated the functional group of GO by EDC/NHS coupling agent and then mixed the ILs and then ultra-sonicated and heated to stirring at the 50 °C temperature. The (NTf_2_^−^)-containing ILs gained hydrophobic character, and then the synthesis was done in the organic phase using dicycohexyl carbodiimide (DCC) coupling agents [75]. Similarly, Liu et al. synthesized the amine-functionalized ILs from N-(3-aminopropyl)-3-decylimidazole and 1-bromodecane in ethanol solvent to heating in mechanical stirring. Further, GO-ILs hybrid nanocomposites were synthesized through grafting between GO and ILs. The prepared composites can be employed for anti-corrosive applications. The preparation of GO-ILs hybrids nanocomposites is displayed in Figure 8A. SEM and TEM images show the occurrence of imidazole ring on the GR sheet in GO-ILs and have a smooth surface with some surface cracks and transparent and thin structure. Moreover, the scanning probe microscope (SPM) image shows the flake framework without any aggregation effect. These results also suggest that GR was exfoliated in the very thin layer having a thickness of 2.82 nm. In addition, the stability of GO-ILs nanocomposites was evaluated through the Raman spectra analysis with different time frames. It indicates that up to 20 h, there were observed no changes in absorption peaks. SEM, TEM, and SPM images of GO-ILs and stability study of the GO-ILs in aqueous solution using the Raman spectrum is shown in Figure 8B [76]. However, the reduced form of GO was utilized by Zhu et al. for the fabrication of gas sensors. They deposited the GO solution on a circular area of filter paper, where GO was reduced by the vapors of hydrazine hydrate at 90 °C for 3 h. Further, RGO was treated with the different ILs, such as (BMIM)(NTf_2_^−^), (BMIM)(BF_4_), (BMIM)(PF_6_), and (BMIM)(OTF) in acetonitrile solution. The preparation of paper-supported RGO-ILs-based flexible sensor array is shown in Figure 8C. The functionalization of ILs effectively influenced the semiconducting property of the RGO, which enhanced their electro-conductive response. The fabricated sensor array is flexible and could effectively detect the volatile organic compound at a very low level [77].

**Figure 9:**
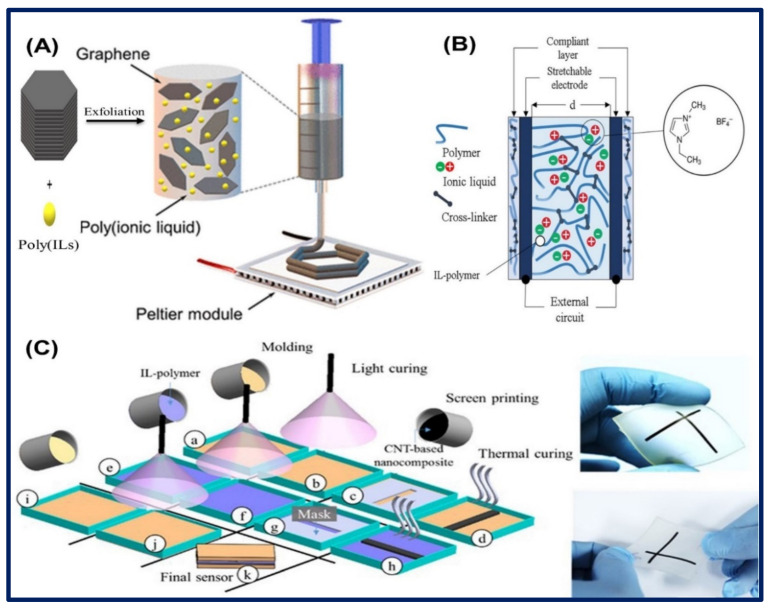
Schematic illustration of the (**A**) freeze 3D printing process. Reprinted with permission from Ref. [78]. Copyright 2020, American Chemical Society. (**B**) Cross-linked ILs/polymer sandwiched between two CNT/polymer electrodes and covered with compliant polymer layers and the chemical structure of (EMI)(BF_4_) used as an IL. (**C**) Left: Schematic of a hybrid manufacturing process including molding and casting (a, e, i), photocuring (b, f, j), screen printing (c, g), and thermal curing (d, h) to fabricate the suggested sensor. Right: fabricated stretchable sensor. Reprinted with permission from Ref. [79]. Copyright 2016, AIP publishing.

Recently, an IL-functionalized GR nano ink was prepared by Tran et al. for electrochemical applications. They did the ultrasonic exfoliation of graphite in IL either poly(1-vinyl-3-ethylimidazolium bromide) or poly(1-vinyl-3-butylimidazolium chloride) aqueous solution. The resulted solution was further centrifuged to increase the concentration of GR nanosheet ∼5.0 mg/mL in IL solution. In this case, IL stabilizes the exfoliation of the graphite layer as well as improves the conductivity of nanomaterials. The freeze 3D printing method was used to fabricate a 3-dimensional layer-by-layer structure of GR nano ink and rapidly dried through the cryogenic process. A schematic of the freeze 3D printing process is displayed in Figure 9A [78]. In another study, Vatani et al. constructed a flexible and stretchable sensor using ILs and CNTs. They prepared ILs/polymer matrix by casting a mold from (EMIM)(BF_4_) mixed with the prepolymer matrix, which was further cured for polymerization. On the other hand, a stretchable electrode of polymer nanocomposites with MWCNTs was also constructed, which serves as a working electrode. In the next step, the ILs/polymer matrix was placed between two MWCNTs/polymer electrodes to make a sandwich assay, where the CNTs considerably enhanced the conductivity of nanocomposites. The fabricated sensor electrode has a width × length × thickness of 1.0 mm × 30.0 mm × 200 µm via layer-by-layer deposition of ILs/polymer nanocomposites, including molding and photocuring at 100 °C for 10 min. The fabricated sensor is effectively applied for the determination of change in pressure. A schematic of a cross-linked ILs/polymer sandwiched between two CNT/polymer electrodes and hybrid manufacturing process is illustrated in Figure 9B,C [79]. 

## 3. Properties of Ionic Liquids 

At the beginning of the 20th century, ethyl ammonium nitrate ILs (EtNH_3_)(NO_3_) (m. p. 12 °C) have been synthesized by using a combination of concentrated nitric acid and ethylamine [44]. After extensive improvements, researchers around the globe succeeded in synthesizing the binary ILs. Nowadays, a large number of ILs are synthesized since the tenability of cationic and anionic species is quite easier. ILs, such as (BMIM)Cl, ethyl-ammonium nitrate, etc., can be synthesized via the one-step method [80].

Functionalization of ILs improves the surface properties of CNMs [81]. In addition, they control the synthesis of nanoparticles as well as prevent agglomeration. In contrast, ILs efficiently serve as a reducing agent for GO, converting it into highly conductive RGO. The improved conductivity, functionality, and high stabilizing power are better aspects for the utilization of ILs in electrochemical analytics. Although the stabilizing potential of the ILs has been due to the low interface energy, their diverse hydrophilic and hydrophobic properties, bulk size with alkyl chain, the functional groups of cationic and anionic moieties, and the absorption on the matrix surface and may result in the high stability of nanocomposites [82,83]. Moreover, the charged bulky ILs create the steric hindrance to affect the surface morphology. The key advantages of the improved characteristics of hybrid nanocomposites result in the enhancement of the performance of the sensors electrode, including sensitivity, detection limit, stability, compatibility, and reproducibility. Furthermore, the high compatibility of the ILs with enzymes gives the efficient sensors comparatively. They enhanced the electrocatalytic activity and fabrication simplicity. However, the proper combination of ILs and CNMs could be utilized for the label-free catalytic sensors. There have been several electro-catalytic sensors reported for the detection of glucose, dopamine, amino acid, and few other analytes. In addition, the recovery and reusability of nanomaterials help in the fabrication of cost-effective biosensors [1,84].

### 3.1. Electrochemical Properties of Ionic Liquids

ILs are well studied because of their superior electrochemical characteristics and are defined by their electrochemical conductivity, potential window, viscosity, and stability. They possess high conductivity ranges from 0.1 to 20.0 mS/cm, low vapor pressure, high thermal stability, and solubility. The viscosity of ILs has a significant effect on the solution due to its rate of mass transport. Furthermore, a combination of large cation and anion may result in the high internal friction of liquids, which causes the high viscosity of ILs. Moreover, the decrease in viscosity is notable by increasing temperature, presence of impurities, and addition of solvents [4,85]. The moderate viscose nanomaterials could serve as a better binder on the electrode surface, which may effectively increase the immobilization of biomolecules, which results in highly sensitive biosensors. Besides, high viscosity could slow the electron transport rate at the electroactive surface, leading to low electrochemical response [85]. On the other hand, the biocompatibility of ILs towards the biomolecules also favors the immobilization of biomolecules, and in few cases, they actively improve the functionality of biomolecules, which results in highly sensitive biosensors with excellent performance [86]. Subsequently, ILs have the potential to replace the conventional electrolytes used in electrochemistry due to low volatility and wide temperature sustainability. The high conductivity and potential window of ILs are useful in electrochemical studies and have wide applicability in electrochemical sensors and biosensors and so on [87]. The conductivity, electrochemical potential window, and viscosity of some ILs are listed in Table 1 [88,89,90].

**Figure 10:**
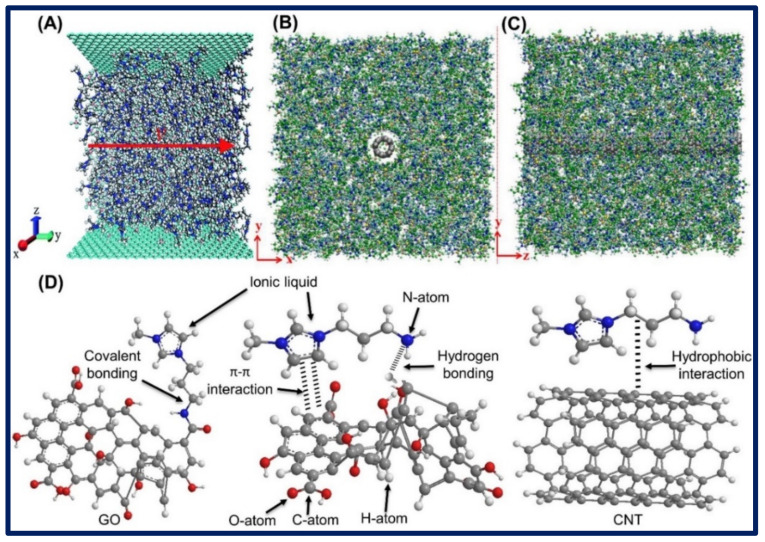
(**A**) The initial structure of the ensemble includes cubic bulk RTILs droplet and GR nanochannel. Reprinted with permission from Ref. [91]. Copyright 2018, Royal Society of Chemistry. (**B**,**C**) The typical equilibrium snapshots (including the top and side views) for the ILs mixture of equimolar (EMIM)(BF_4_) and (BMIM)(PF_6_) around the CNTs. Reprinted with permission from Ref. [92]. Copyright 2020, American Chemical Society. (**D**) Covalent and non-covalent interaction of ILs with CNMs.

Conversely, in another study, molecular dynamic (MD) simulation was used to investigate the flow-induced voltage of the (EMIM)(BF_4_) flowing over the two parallel, single-layer GR sheet nanochannels with the dimension varies from 1.0 to 25.0 nm^2^. The MD simulation calculated the maximum flow-induced voltage to be 2.3 µV. The study revealed that the nanochannel diameter and nature of fluid viscosity and temperature greatly affected the flow-induced voltage. It was found that when the diameter of the nanochannel increased from 1.0 nm to 5.0 nm, the flow-induced voltage also increased from 1.9 to 2.1 µV. However, in the case of increment of nanochannel from 1.0 to 25 nm^2^, it decreased from 2.3 to 2.1 µV. Moreover, the enhancement of applied acceleration and temperature increased the flow-induced voltage. The initial structure of the ensemble, including cubic bulk RTILs droplet and GR nanochannel, is illustrated in Figure 10A [91]. Similarly, Wang et al. carried out MD simulation to study the dynamics, structure, and H-binding of the equimolar mixture of two ILs (EMIM)(BF_4_) and (BMIM)(PF_6_) around the SWCNTs with distinct diameters. Studies revealed that (PF_6_^−^) anion shows stronger H-bonding with the imidazolium cations as compared to (BF_4_^−^) anion. However, the increment in H-bonding was observed with CNTs and both anions, where H-bonding increased with an increase in the diameter of CNTs. Nevertheless, it was found that the (PF_6_)^−^ containing ILs aggregated more around the large CNTs, while (BF_4_)^−^ have less tendency for such property. The typical equilibrium snapshots of the ILs’ mixture of equimolar (EMIM)(BF_4_) and (BMIM)(PF_6_) around the CNTs are shown in Figure 10B,C [92]. 

### 3.2. Bonding of Ionic Liquid-Carbon Hybrid Nanomaterials

Studies showed that during synthesis, functional ILs attached to the CNMs by the covalent and non-covalent interactions or electrostatic force of attractions, including hydrogen bonding, π–π interactions, ion–dipole, dipole–dipole, and van der Waals, ion-induced dipole, and permanent dipoles interactions as well as dispersion forces. The interaction of CNMs with ILs depends upon the functionality and availability/nature of cations and anions of ILs. These interactions or physical/chemical forces brought about several improvements in the properties of CNMs, such as conductivity, hydrophilicity, hydrophobicity, stability, etc. [93,94]. Covalent and non-covalent bonding of ILs with CNMs are shown in Figure 10D.

## 4. Ionic Liquids–Carbon-Based Functional Nanomaterials in Biosensing Applications 

ILs-integrated CNMs are well studied in electrochemical sensors and biosensors since easy preparation of hybrid nanomaterials and their enormous properties attracted their utility in the fabrication of biosensors. Biosensors are analytical devices that are combined with a biological component integrated with the physical transducer, which generates the quantitative or semi-quantitative signal response and detects the target analyte. In the stepwise electrochemical biosensor fabrication, the surface of the electrode is fabricated by the metal nanoparticles, metal oxides, metal sulfides, carbon-based nanomaterials (GR, GO, RGO, QDs), metal dichalcogenides (MoS_2_, WS_2,_ etc.) or nanocomposites [95,96,97]. Further, the target-specific biomolecules, either antibody or antigen, are immobilized to bind with selective analytes, which demonstrates the specificity of the biosensors. The antibody or antigen interacts with the target analyte that displays antibody–antigen chemistry, which generates the measurable signal for detection of the analyte. However, in the case of catalytic detection, selective enzymes are used to modify the electrode surface to catalyze the target analytes. The release of electrons is measured as a response to find the concentration of the target analyte. [98,99]. 

Several nanomaterials are being utilized to construct the biosensor, but the ILs-functionalized carbon nanocomposites get considerable attention in the development of the electrochemical biosensor [100,101]. There are numerous biosensors reported so far, and of these, electrochemical biosensors are highly sensitive and selective tools for detection of various analytes, including antigen, antibody, protein, DNA, RNA, metabolites, ions, etc. Moreover, they have high reproducibility, a wide detection range, and ultra-low LOD capacity, cost-effectiveness, disposable, long-term stability, user-friendly, and reusability [102]. Consequently, they could be implanted as flexible and wearable biosensors that could be implemented as the point-of-care (POC) biosensors for on-site diagnostics [103,104]. Nowadays, the development of ILs-CNMs-based, highly sensitive electrochemical sensors and biosensors continuously attract researchers and is continuously growing, which will become a hot topic of research in the future scenario. Very recently, there have been several sensors reported that were fabricated of the nanocomposites of ionic-liquid-functionalized carbon nanomaterials with/without metal nanoparticles for the detection of pharmaceutical drugs [105], mycotoxin [106,107], bisphenol [108], amino acid [109], and food additives [110]. In another report, ILs-functionalized metal-organic framework (MOF) based on a highly sensitive biosensor was reported for the detection of cancer biomarkers [111]. 

There are several IL-CNMs-based electrochemical sensors and biosensors reported that have remarkable performance. The information of IL-CNMs hybrid nanocomposites-based electrochemical sensors and biosensors for the detection of numerous analytes is summarized in Table 2.

**Figure 11:**
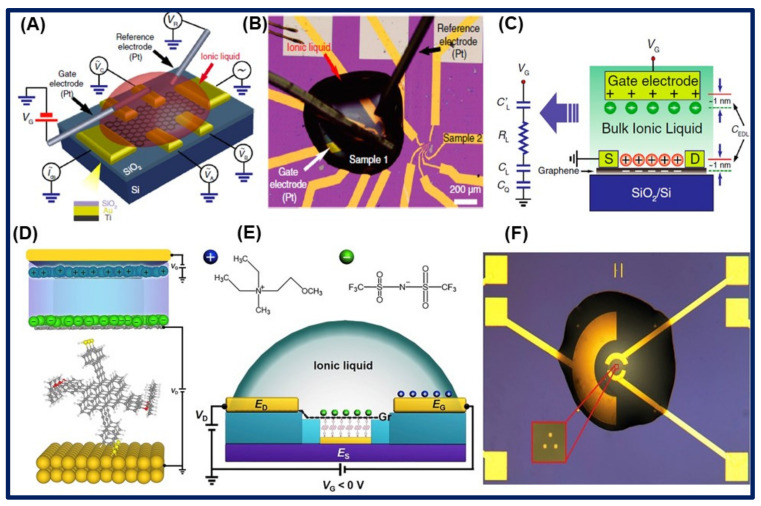
Electric double-layer GR devices. (**A**) A schematic representation of a device, including the bias configuration used in the electrical measurements. (**B**) Optical microscope image of an actual device. (**C**) A schematic cross-section of GR EDLTs, together with the equivalent electrical circuit describing its operation. Reprinted with permission from Ref. [149]. Copyright 2011, PNAS Publishing. (**D**–**F**) Schematic illustration for the setup of the device with a vertical IL gate through GR layer to SAMs and molecular structures for DEME^+^ cation and TFSI^−^ anion. Reprinted with permission from Ref. [150]. Copyright 2019, Elsevier.

Recently, field-effect transistor (FET) has also emerged as a promising sensor for the detection of target analytes with high sensitivity and specificity. They are mainly containing the source, drain, and gate channel. For a long time, GR has been the utmost functioning nanomaterial to fabricate the FET biosensors. Inaba et al. fabricated an IL-GR FET for sensing application. FET channel of the width and length of 50 µm × 20 µm was constructed by the chemical vapor deposition of a thin layer of a GR sheet on the Cu foil surface, and further chemical etching through oxygen plasma was done. After that, GR sheet was deposited on the gold surface, and then researchers immobilized the ionic liquids (EMIM)(BF_4_) or the mixture of (EMIM)(BF_4_) and poly(ethyleneimine) (PEI) to develop the source channel and gate, respectively, where ILs cover the GR surface. ILs behave as an insulator and catching agent for a selective target. When the gate voltage is applied, it causes the generation of a very thin, electric double layer, which allows the working of FET at very low voltage as compared to the SiO_2_ gate layer. A curve of source-drain current (I_ds_) vs. gate voltage (V_g_) was evaluated to detect the specific target [148]. Similarly, Ye et al. fabricated electric double-layer devices of mono, bi, and trilayer graphene on the SiO_2_ substrate enabled with the ionic liquids gate. The conductivity of the mono layer GR is saturated, while bi and trilayer fall in the high energy band, which indicates enhancement of the high-density transport property of graphene. A schematic illustration of an ILs-GR device, including the bias configuration used in the electrical measurements, an optical microscope image of an actual and cross-section of our graphene EDLTs, together with the equivalent electrical circuit describing its operation, are shown in Figure 11A–C [149].

In another context, Famili et al. fabricated an electrochemical FET device by using diethyl methyl(2-methoxyethyl)ammonium bis(trifluoromethylsulfonyl)imide (DEME-TFSI) IL on the single-layer GR, which was deposited on the self-assembled monolayers (SAMs) and gold channel surface to make a source channel. Schematic for the setup of the device with a vertical IL gate through the GR layer is illustrated in Figure 11D–F [150].

In this section, we thoroughly discuss the functional ILs-incorporated carbon hybrid nanomaterials-based electrochemical sensors and biosensors for the detection of cancer, and cardiac biomarkers, immunoglobulins, and neurotransmitters. In addition, the detection of a few other markers, such as glucose, choline, acetylcholine, cholesterol, uric acid, ascorbic acid, etc., is also discussed. Last, further improvements and their respective limitations are also highlighted.

### 4.1. Electrochemical Analytic for Cancer Biomarkers

Currently, cancer has emerged as a deadly disease caused by the uncontrolled growth of cells in the body. Cancer is the second utmost cause of mortality worldwide. WHO estimated that approximately 10.0 million deaths were caused by cancer in the year 2020, with breast, lung, liver, stomach, colon, and rectum cancers being the leading cancers that affect a large mass of the population globally [151,152]. An early diagnosis of cancer has great importance and has the potential to diminish the mortality rate as well as increase the survival rate [153]. Cancer biomarkers that are disease indicators are categorized as proteomic, transcriptomic, metabolomic, and genomic, where the biomarkers are targeted to be detected through the biosensor, which is highly sensitive and selective against disease-specific biomarkers that indicate the disease stage. Therefore, screening of cancer at the early stage becomes more significant to control the disease progression as well as for treatment [154,155]. In this perspective, numerous highly sensitive, specific, and reproducible biosensors have been reported for the diagnosis of cancer biomarkers [156,157]. For instance, Huang et al. reported an aptamer-based biosensor for the detection of carcinoembryonic antigen (CEA) biomarker at femtogram concentration with high sensitivity. The detection strategy was based on the GQDs-ILs-Nf (Nafion) film and DNAzyme-assisted signal amplification method, which was depending upon Pd^+2^ concentration. They developed a specific aptamer of CEA containing hairpin DNA. When CEA meets hairpin DNA, it cleaves and interacts with the substrate chain. Further, Pb^+2^ ion bound to substrate chain containing CEA-labeled hairpin DNA, where they broke the substrate chain to produce the ssDNA. Subsequently, Pb^+2^ played an essential role in the cleavage of the substrate chain, and cleavage was proportional to the concentration of Pb^+2^. This ssDNA was captured on the methylene blue-labeled DNA-immobilized electrode surface of GQDs-ILs-Nf that produced the electrochemical signal. The quantification of the signal revealed that the biosensor has large detection ranges from 0.5 fg/mL to 0.5 ng/mL with the LOD of 0.34 fg/mL. The proposed biosensor could be successfully applied in the serum analysis [112]. In another study, Zhao et al. reported an IL- and RGO-based voltammetric biosensor for the detection of CEA. They fabricated an electrode by electrochemical reduction of GO into RGO on GCE, followed by the electro-polymerization of functionalized poly(indole-6-carboxylic acid) (PICA) onto the RGO surface. Further, chitosan containing (BMIM)(PF_6_) IL solution was deposited on the modified electrode surface, where the IL improved the conductivity and prevented the agglomeration of the matrix. In addition, a layer of AuNPs was deposited on IL-RGO-PICA to enhance the conductivity, surface area, and stability of electrodes, which made them more sensitive. DPV measurement revealed that the sensor has excellent sensitivity, reproducibility, accuracy, and wide detection ranges from 0.02 to 90.0 ng/mL with a LOD of 0.02 ng/mL. They compared the biosensor with the Fe_3_O_4_@SiO_2_-based amperometric sensor and pointed out that the developed voltammetric biosensor has superior performance with a low detection limit and high sensitivity. They further validated the biosensor by comparative assessment with the enzyme-linked immunosorbent assay (ELISA) and found satisfactory results [113]. Similarly, Liu et al. proposed a sandwich-type voltammetric biosensor for simultaneous diagnosis of alpha-fetoprotein (AFP) and CEA as low as the nano-gram concentration. They utilized the amine-terminated IL (NH_2_-IL) for the fabrication of the electrode surface, where NH_2_-IL functionalized on the GO by epoxy ring-opening reaction, and further, it reduced the GO to RGO. In addition, the IL prohibited the agglomeration of nanocomposites matrix and provided the amine functionality, which significantly introduced the high immobilization of AuNPs. The anti-AFP antibody or anti-CEA antibody were immobilized on a modified sensor surface that specifically binds to AFP or CEA antigen to serve as a biosensing platform. On the other hand, they synthesized the two nanocomposites, i.e., chitosan containing Prussian blue-gold nanoparticles (PB-CS-Au NPs) and cadmium sulfide-gold nanoparticles (Cd-CS-AuNPs), and were modified by anti-CEA and anti-AFP antibody, respectively, and functioned as distinguishable signal tags. The binding of target antigen between two specific antibodies displayed antibody-antigen chemistry, which generated the detectable signal to analyze the AFP and CEA simultaneously [114].

**Figure 12:**
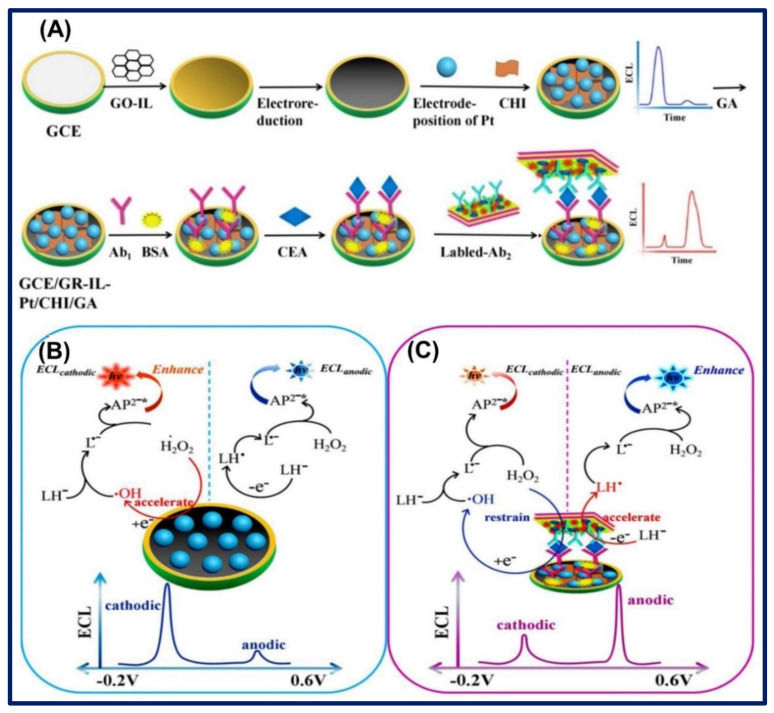
(**A**) The fabrication strategy of ratiometric ECL biosensor. The possible ECL mechanisms of the biosensor without (**B**) and with (**C**) Ti_3_C_2_ MXenes-AuNPs-Ab2 nanoprobe. Reprinted with permission from Ref. [115]. Copyright 2020, Elsevier.

Very recently, Shang et al. developed the GR, ILs, and platinum nanoparticles (GR-ILs-PtNPs)-based nanocomposites of sandwich electrochemiluminescence (ECL) assay for the ratiometric determination of CEA in serum samples. Firstly, the primary antibody (Ab1) was immobilized on GR-ILs-Pt, while the secondary antibody (Ab2) was immobilized on Ti_3_C_2_-MXenes-AuNPs hybrid nanocomposites. These Ab1- and Ab2-immobilized nanocomposites generated the cathodic and anodic ECL signals through capturing of CEA between Ab1 and Ab2, respectively. Subsequently, the anodic signal increased, while the cathodic signal decreased; as a result, the ratio of anodic and cathodic ECL signal (ECL_anodic_/ECL_cathodic_) increased, which was employed in the ratiometric estimation of CEA. The calculated LOD of ECL assay was 34.58 fg/mL with high accuracy, sensitivity, and specificity, as shown in Figure 12A–C [115]. Another ECL technique was employed by Wang and the group for the determination of CEA biomarkers responsible for various cancers. They utilized the nanocomposites of IL-functionalized GR with highly porous PtNPs (GR/IL/pPt) for fabrication of electrode surface, where the working of the biosensor was based on the measurement of cathodic ECL signal of 3-aminophthalate (luminol). They examined that the signal intensity of the GR/IL/pPt-based cathodic ECL was three times greater than the GR/IL-based electrode. They suggested that the enhancement of signal was due to the large surface area and porous nature of Pt. However, a higher ECL signal was obtained due to the additional excitement of luminol, where the luminol reacted with the reactive oxygen species (ROS), which was generated by the catalytic reduction of H_2_O_2_ through the GR/ILs/pPt electrode. The proposed immunosensor has high sensitivity and reproducibility, and the LOD of 0.0003 fg/mL was observed in clinical specimens [116].

Recently, Wei and coworkers reported the electrochemical sandwich immunoassay for the diagnosis of prostate-specific antigen (PSA) through the estimation of catalytic activity of H_2_O_2_. They fabricated the sensor surface by nanocomposites of AuNPs with cobalt sulfide and GR (Au-CoS/GR), where the primary antibody (Ab1) was immobilized to introduce the specificity in the immunosensor. Further, the second nanocomposites were synthesized by amine-functionalized, mesoporous CeO_2_ labeled with toluidine blue (TB/M-CeO_2_) and incorporated with IL-doped carboxymethyl chitosan to form (TB/M-CeO_2_/CMC/ILs), at which secondary antibody, anti-PSA antibody (Ab2), was immobilized. The M-CeO_2_ has a high surface area, uniform size distribution, and excellent water dispersibility, whereas CMC/ILs enhanced the electron transfer ability and high absorption efficacy for TB. The study showed that the PSA concentration significantly influenced the electrochemical signals, where the DPV signals were increased for TB/M-CeO_2_/CMC/ILs, but the amperometric signal decreased for Au-CoS/GR. The voltammetric and amperometric analyses were done to detect the PSA, and the linearity and LOD of the sensor were calculated to be 0.5 pg/mL to 50.0 ng/mL and 0.16 pg/mL, respectively [117]. 

Similarly, PSA was diagnosed at nanogram concentration through a label-free voltammetric biosensor consisting of CS-GR-IL-Fc-AuNPs-based 3D nanostructures fabricated through the cryogenic method reported by Choosang and coworkers. Herein, they cast the nanocomposites containing chitosan, GR, and (BMIM)(TFSI) labeled with ferrocene on GCE, and further freezing and thawing were done to generate a 3D nanoporous structure. AuNPs were immobilized on the porous surface to enhance the conductivity and increase the electrochemical response. The porous structure improves the loading of AuNPs, which results in the high loading capacity of biomolecule and enhances the sensitivity of the biosensor. They also compared and found that the sensitivity of the CS-GR-IL-Fc-AuNPs-anti-PSA was 4.3-, 2.7-, and 1.4-fold higher than the CS-Fc-AuNPs-anti-PSA, CS-IL-Fc-AuNPs-anti-PSA, and CS-GR-Fc-AuNPs-anti-PSA, respectively. The electrode has the advantage that could be regenerated by washing with HCl (pH 2.0) and reused up to 48 individual measurements, and it has stability up to 20 days with the >90.0% of sensitivity and high reproducibility. As compared with the ELISA, the performance of the biosensor was observed to be excellent, which makes them a suitable candidate in clinical applications. In addition, biosensor has high selectivity against PSA even in the presence of other biomarkers, such as human serum albumin (HAS), AFP, and CEA [118]. In another report, a microRNA-34a biomarker as an indicator of cancer, Alzheimer’s, and cardiovascular disease was detected by Kesici and coworkers through an impedimetric biosensor. They utilized the (BMIM)(PF_6_) IL to modify the chemically activated pencil graphite electrodes (PGEs). Herein, the pencil graphite electrodes were chemically functionalized using the suitable coupling agent (1-Ethyl-3-(3-dimethyl aminopropyl)carbodiimide/N-hydroxy succinimide) (EDC/NHS) to introduce the acid functionality on the surface. Authors found that the surface area of the biosensor was increased by using IL, which improves the immobilization of biomolecules. Further, the biosensor surface was labeled with the DNA-RNA hybrid conjugates (hybridization of miRNA-34a-specific DNA probe and miRNA-34a target) to selectively target the microRNA-34a. The biosensor has acceptable detection of concentration, ranging from 2.0 to 10.0 µg/mL with LOD of 0.772 and 0.826 µg/mL in PBS and fatal bovine serum, respectively [119]. Similarly, those authors utilized the same matrix to the determination of microRNA-34a through voltammetry detection technique in real samples with the LOD of 0.56 and 0.40 µg/mL in buffer medium and diluted artificial serum, respectively. In addition, the sensor has high specificity towards miRNA-34a even in the presence of miRNA-155, miRNA-660, and miRNA-34a MM [120].

Human papillomavirus (HPV) is a common viral infection transmitted through sexual contact and, later on, develops into cancer. In this context, Farzin et al. reported the highly sensitive and specific electrochemical biosensor for diagnosis of HPV16 with LOD of 1.3 nM in clinical samples. In this study, they synthesized the nanostructured amine-terminated IL with RGO and immobilized it on MWCNT-modified GCE. Furthermore, the DNA probe was loaded on a modified electrode surface via a glutaraldehyde cross-linker. These DNA probes hybridize with the HPV-16 DNA strand in the presence of anthraquinone-2-sulfonic acid monohydrate sodium salt as they act as a redox-active substrate, resulting in a measurable signal that could be used to identify HPV-16 in clinical samples [121]. 

### 4.2. Electrochemical Analysis of Cardiac Biomarkers

**Figure 13:**
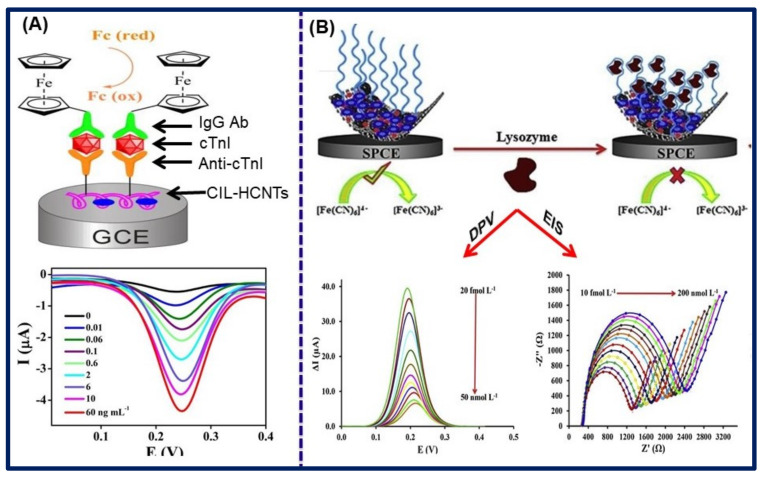
Schematic illustration of the (**A**) fabricated electrochemical immunosensor for cTnI. Reprinted with permission from Ref. [121]. Copyright 2018, Elsevier. (**B**) Step-by-step preparation of SPCE/Amino-RGO/ILs/Amino-MSNs/APT. Reprinted with permission from Ref. [145]. Copyright 2019, Elsevier.

Cardiovascular disease is considered a major disease not only concerned with the heart problem but also as it relates to blood vessels. Occurrence could be influenced by several factors, including genetic disposition, high blood pressure and cholesterol, diabetes, obesity and overweight, smoking, and stress [158]. Several biomarkers, such as cardiac troponin I (cTnI), troponin T (cTnT), and troponin C (cTnC), myoglobin, C-reactive protein (CRP), creatine kinase (CK), etc., have been found responsible and recommended to be checked for the diagnosis of cardiac disease [159]. The early and rapid diagnosis of cardiac disease is very important for increasing the patient survival rate as well as the successful prognosis of the diseases [160,161]. In this context, Yan et al. explored the efficiency of ionic liquid-based nanocomposites for the diagnosis of cardiac troponin I (cTnI) by the electrochemical sandwich assay. In this work, carboxy terminated methyl-3-methylimidazolium chloride mixed with helical carbon nanotube (CIL-HCNTs) through an ultra-sonication method to construct a sensor surface, where they capture via π–π and π–cation interaction. Carboxyl functionality favors the high loading of anti-cTnI antibodies to increase the selectivity and sensitivity of the biosensor. To achieve the sandwich assay, they labeled the secondary antibody onto carboxyl functionalized ferrocene (IgG-Ab-Fc-COOH), which binds to cTnI/BSA/anti-cTnI/CIL-HCNTs/GCE primary electrode. They revealed that the peak current density increases gradually with the increase of cTnI concentration, which occurs due to detachment of ferrocene from the assay at the time of detection. The biosensor was highly sensitive, and detection range of concentrations was from 0.01 to 60.0 ng/mL with a low LOD of 0.006 ng/mL along with acceptable detection capacity in human serum samples, as shown in Figure 13A [122].

In another report, Shen et al. demonstrated a label-free voltammetric biosensor for the diagnosis of cTnI. Herein, they also utilized the helical carbon nanotubes (HCNT) and dialdehyde-functionalized ionic liquid (DIL) nanocomposites for the construction of the biosensor. Similarly, HNCT was bounded to IL through π–π and π–cation bonds. Moreover, HCNT properties could be efficiently modified through chemical methods. In addition, the lattice defect of CNT can significantly enhance electrochemical performance. However, dialdehyde functionality facilitates the immobilization of antibodies on the sensor surface, which improves the sensitivity of the biosensor. The authors calculated the linear detection range of the biosensor to be 0.05–30.0 ng/mL and LOD of 0.03 ng/mL. The sensor possesses good reproducibility, sensitivity, and shelf life up to 15 days when stored at 4 °C [123]. 

### 4.3. Electrochemical Investigation of Immunoglobulins

The immune system of the body responds to generating the immunoglobulins known as antibodies to protect against harmful foreign substances, such as an antigen. They are mainly immunoglobulin G (IgG), immunoglobulin M (IgM), immunoglobulin A (IgA), and immunoglobulin D (IgD) [162]. The quantitative measuring of immunoglobulin is the key to determining the immune response, diagnostics, and health condition of the body against foreign substances [163]. There have been numerous immunosensors reported to detect immunoglobulins for assisting diagnosis [164]. For instance, Shen et al. detected the immunoglobulin G (IgG) through the voltammetric sensor. They developed the immunosensor of amine-terminated carbon nanotube (CNT-NH_2_) and aldehyde-functionalized IL-CHO nanocomposites followed by the immobilization of capture antibody. Here, IL enhances the electron transport mobility and prevents the leaching of electrode surface, which could improve the immunosensor performance. They examined that the LOD of the immunosensor was 0.02 ng/mL with the linearity of 0.1–15 ng/mL. The sensor could perform satisfactorily in PBS as well as in clinical samples [124]. Similarly, these authors developed the electrochemical sandwich immunoassay using a similar matrix with some modifications for the diagnosis of IgG in the clinical sample. Herein, they utilized the nanocomposites of acid-functionalized CNT and ferrocene-labeled IL-CHO (CNT/Fc-IL-CHO) to construct the sensor surface where the aldehyde functionality serves as binding sites for secondary antibodies and ferrocene as a signal probe. Moreover, the primary antibody was immobilized on the gold electrode using chitosan and glutaraldehyde cross-linker. This assay has good reproducibility, long-term stability, and an excellent detection range of 0.05–30 ng/mL with LOD of 0.01 ng/mL, which is 40-fold greater than other ferrocene-labeled immunosensors for IgG [125]. In a recent study, Shen et al. introduced the label-free immunosensor for diagnosis of IgG using matrix containing Nafion and aldehyde-functionalized IL with ferrocene. Nafion is used as a conducting solid electrolyte polymer to form a membrane on the sensor surface, which allows the transfer of ions between the electrodes. The authors suggested that the reported sensor has exceptional ability to detect the IgG at a high sensitivity and low LOD of 0.03 ng/mL in the biological sample [126].

A high level of CRP indicates various health-related problems, such as cardiovascular and inflammatory disease. Recently, a C-reactive protein (CRP) was detected by a label-free electrochemical immunosensor in serum. Dong et al. constructed an immunosensor of zinc oxide, porous carbon structure, and IL, which displays high catalytic properties. Porous carbon matrix (PCM) is synthesized from the pyrolysis of mixed ligand metal-organic framework (MOF) in a controlled manner. The porous nature of carbon favors a high loading capacity. Moreover, ZnO is less toxic and highly biocompatible and chemically stable. Besides this, the semiconducting property of ZnO limited its utilization in an electrochemical sensor. The existence of O-vacancies, C-O, and Zn-O is favorable to improving the ionic and electrical conductivities of ZnO/MPC. On the other hand, SEM analysis shows that some rod-shaped ZnO-PCM is also formed, which retains the bioactivity and improves the biomolecule immobilization. The immunosensor has excellent performance and ultra-low LOD of 5.0 pg/mL [127]. In another context, Xia et al. reported a molecularly imprinted polymer-based electrochemical biosensor for the detection of bovine serum albumin (BSA). The authors performed the functionalization of (BMIM)(PF_6_) onto GR followed by molecular imprinting of pyrrole and BSA as a monomer and template, respectively, through electrochemical polymerization to construct MIP. The biosensor has good reproducibility and stability up to 15 days when stored at 4 °C [128]. 

### 4.4. Detection of Neurotransmitters

Dopamine (DA) is a neurotransmitter that controls the physical functions of the body and is used to transmit messages into nerve cells under the nervous system. It develops the ability to think and plan by striving, focusing, and function. High or too low levels of dopamine can lead to serious health issues, like Parkinson’s disease and schizophrenia [165,166]. The early diagnosis of DA may lead to control and prognosis of the related disease caused due to alteration of concentration of neurotransmitter. In this concern, several sensors are reported for the detection of neurotransmitters [167]. For instance, Li et al. reported the voltammetric sensor for the detection of dopamine at very low concentrations. Herein, they functionalized the 1-butyl-3-methyl imidazole hydrobromide IL on GO via electrostatic interaction in a one-step reaction, and further AuNPs were incorporated onto GO-IL nanocomposites. The introduction of IL could efficiently inhibit the agglomeration of GO and enhance the conductivity of the matrix. Moreover, AuNPs provide a high surface area, high electro-catalytic property, stability, and biocompatibility. Authors concluded that the charge transfer-resistant property of the GO was found to be 1032 Ω, but it dramatically decreases to ~9-fold after functionalization with IL and a further ~28-fold decrease after the introduction of IL and AuNPs. This property revealed that the GO-IL-AuNPs have excellent conductivity and catalytic properties for dopamine. The reported sensor has high feasible nature and has a low LOD of 2.3 nM as well as satisfactory performance in the real samples [129]. Similarly, dopamine was detected by voltammetric biosensor of IL functionalized GR and CQDs with the LOD of 30.0 nM. The functionalization of IL tends to increase the dispersibility and stability of GR and reduce its agglomeration behavior. Moreover, the carboxylic functionalization of CQDs may attribute to interaction with dopamine, which results in the high redox response. Furthermore, the sensor possesses excellent specificity for DA even in the presence of similar substances, such as epinephrine and 3-methoxy phenol [130]. 

Recently, Kunpatee et al. simultaneously detected dopamine (DA), ascorbic acid (AA), and uric acid (UA) through the electrochemical measurement. They modified the sensor by GQDs-IL nanocomposites, where IL enhanced the electrochemical response; however, adding GQDs onto the (BMIM)(PF_6_) IL feasibly improves the electro-catalytic property. The biocompatibility and low toxicity of GQDs and IL make it favorable in electrode fabrication, which could be directly implanted as a wearable sensor for on-site diagnostics. In addition, the utilization of GQDs-IL improves the oxidation peak and differentiates the responsive oxidation peak of analytes (DA, AA, and UA) to overcome the peak separation problem of multiple analytes. The proposed sensor could serve in the diagnosis of clinical samples and pharmaceutical products [131]. In another report, Nagles et al. developed a voltammetric sensor for detecting DA and UA in human urine samples even in the presence of AA without any interference. They constructed an electrode by casting of (BMIM)(BF_4_) IL incorporated with SWCNT in chitosan solution. SWCNT has high conductivity, while IL further improves the conductivity and anodic current response. The authors revealed that the anodic peak current of (BMIM)(BF_4_) was higher than the (BMIM)(PF_6_) for DA and UA. They suggested that this outcome is due to the larger size of PF_6_ anion as compared to BF_4_, which hinders the movement of electron transport. Through the LSV study, the LOD of the sensor was found to be 0.16 and 0.17 µmol/L for DP and UA, respectively [132]. Another neurotransmitter, acetylcholine (Ach), and its precursor, choline (Ch), responsible for Alzheimer’s and other diseases, were detected by an electrochemical sensor in a serum sample having LOD of 1.352 and 0.885 nmol, respectively. The sensor was constructed by (AMIM)(TFSI) IL-functionalized GO followed by the immobilization of acetylcholine esterase (AchE) and/or choline oxidase (Cho) for selective detection of the target analyte. The detection of Ch was done by the enzymatic catalytic action via Cho to generate H_2_O_2_ while the Ach catalyzes by the AChE to firstly generate the Ch and further convert it into H_2_O_2_. The oxidation current produced by the H_2_O_2_ can be used to determination of the concentration of Ch and ACh. However, the use of serum samples does not need purification or pre-treatment before the diagnostic process [133]. 

### 4.5. Detection of Glucose

Glucose is a monosaccharide and energy source of the living organism. The oxidation of glucose converts into gluconic acid and releases enormous amounts of energy, which is used to functionalize the metabolic process. However, in any circumstances, when the glucose level increases in the blood either by high consumption or less catalytic activity, it turns to a disease named diabetes. The increase in glucose levels causes serious health problems and is responsible for multiple organ failure, like that of the lungs, kidney, liver, etc., and causes early death [168]. In this regard, several enzymatic and non-enzymatic sensors have been reported. In the enzymatic sensor, glucose oxidase enzymes catalyze the glucose to enable the detection of glucose concentration in blood. However, in a non-enzymatic sensor, appropriate material is fabricated on the sensor surface, which matches the catalytic property against glucose to appropriately catalyze the glucose [169,170]. For instance, Naeim et al. developed a non-enzymatic electrochemical sensor of hybrid nanostructured bimetallic Ni-Pd nanoparticles modified IL-RGO on GCE. The hybrid nanocomposites were synthesized by the immobilization of IL in GO solution in a basic medium. Further, nickel salt and palladium salt were added and reduced to form RGO-IL-Ni-Pd nanocomposites. The NiNPs exhibit high electro-catalytic activity, while PdNPs further improve the catalytic property of the matrix of the sensor. However, IL prohibited the agglomeration of GO and controlled the synthesis of nanoparticles as well. The amperometric detection of glucose showed that the sensor had a wide linear detection range from 0.0002 to 10.0 mM and a LOD and sensitivity of 0.03 µM and 1504.61 mA/mM cm^−2^, respectively [134]. Similarly, Benjamin et al. reported a non-enzymatic electrochemical sensor for the detection of glucose with the detection range from 0.2 µM to 1.8 mM and LOD of 0.79 µM. Herein, they employed the salen type ligand, modified by cobalt nanoparticles, and IL to form a cobalt-salophen-IL complex (salophen–IL = N, N’-(o-phenylene)bis-((3-ethyl-1H-benzimidazole-1-ium-1-yl)methylene hexafluorophosphate) salicylideneimine) and cast on electrochemically RGO (ERGO)-modified SPE to form Co-salophen-IL/ERGO/SPE, where the salophen-IL complex has excellent electro-catalytic activity. Amperometric investigation revealed that the electro-catalytic activity for the glucose was optimum at 0.40 V potential, which generates the high current response. Therefore, the oxidation of glucose at the lower potential makes them highly sensitive and efficient detection tools for the detection of glucose in serum and urine samples. Furthermore, the selectivity of the sensor was tested in several interfering agents, such as AA, DA, and UA, and no such type of interference was found [135].

**Figure 14:**
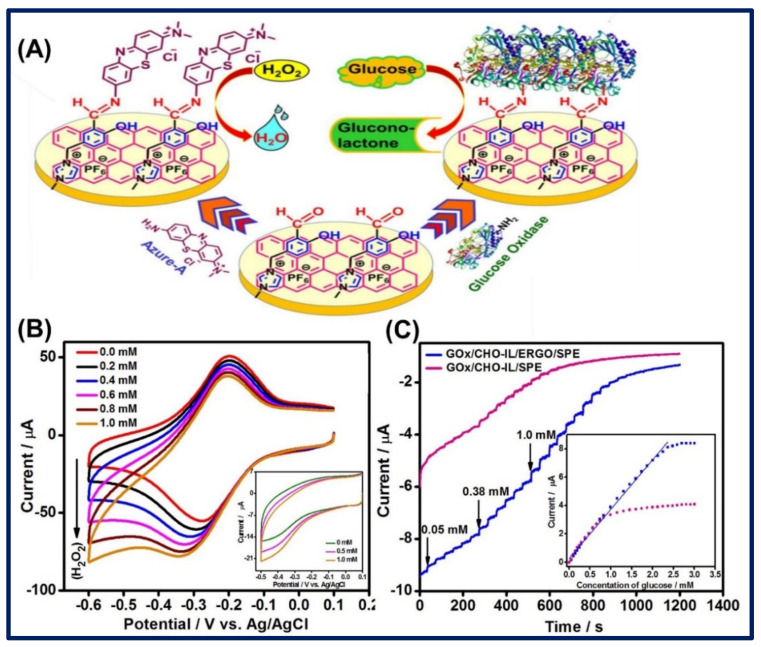
(**A**) Schematic illustration of the electrochemical sensor platform for H_2_O_2_ and glucose detection. (**B**) CVs plot of Azu-A/CHO-IL/ERGO/SPE with increasing concentration of H_2_O_2_. (**C**) Amperometric response for Azu-A/CHO-IL/SPE and Azu-A/CHO-IL/ERGO/SPE on successive additions of 30 μM H_2_O_2_ into the electrolyte. Reprinted with permission from Ref. [136]. Copyright 2018, Elsevier.

Similarly, Manoj and coworkers utilized the ERGO modified by aldehyde functionalized-IL (3-(3-formyl-4-hydroxy benzyl)-3-methylimidazolium hexafluorophosphate) through the π–π staking, followed by the immobilization of glucose oxidase enzyme (GOx) or Azure A organic dye on a modified surface for detection of glucose or H_2_O_2_, respectively. Through the amperometric determination, the optimum potential for catalytic activity was found to be -0.45 V and -0.35 V for GOx and Azure A-based electrodes, respectively. The electrode was highly sensitive and quantitatively calculated to be 17.7 and 133.2 μA/mM cm^−2^ for glucose and H_2_O_2_, respectively, whereas the sensor quickly performed and responded in 3.0 s for both electrodes. In addition, the sensor shows excellent performance and produced measurable peak current even after 100 cycles for GOx-CHO-IL-ERGO electrode; additionally, it has the stability of up to 30 days with losing only 9.0% current response from their initial performance. Therefore, the diagnosis of glucose at the lower potential with high current response and sensitivity through the reported sensor could be a good candidate for clinical application. Schematic illustration of the electrochemical sensor platform and amperometric detection response for H_2_O_2_ and glucose detection is shown in Figure 14A–C [136].

Very recently, Janmee and colleagues reported a disposable, non-enzymatic sensor for the rapid detection of glucose in urine and electrolyte drinks. Herein, they deposited the CuO-IL-RGO nanocomposite on SPE, which was utilized for the quantitative estimation of glucose via chronoamperometric, where the result can be obtained in 20 s. In this detection, there is no need for sample pre-treatment. Furthermore, comparative studies show that the sensor has considerable detection performance as compared to the commercial glucose sensors. Therefore, this could be an alternative to the future scenario [137]. In another study, a label-free, GR-enabled, paper-based electrochemical sensor was reported for the determination of glucose in clinical samples. The nanocomposites materials consisting of cobalt (II) phthalocynin (CoPc) and IL-functionalized GR were utilized to fabrication of sensor. CoPc is metallophthalocycnin, which has high electrocatalytic characteristics towards glucose. However, GR and IL further enhanced the electrochemical property and stability of the sensor. At the 0.7 V operating potential, the LOD was calculated to be 0.64 µM for glucose. This sensor could be successfully utilized for monitoring glucose in serum, white wine, and honey. In addition, they have the potential to turn into POC devices for healthcare monitoring [138]. Recently, Zou et al. reported a voltammetric-based enzymatic sensor for the determination of glucose. Herein, a 3D nanostructured electrode surface was constructed by utilization of imidazolium-based IL immobilized on the GO through covalent interaction via ring-opening reaction. To further improve the catalytic property and electro-conductivity, they utilized the MWCNT, where it was stacked between the GR layers, which enhanced the electro-conductivity and surface area. The high surface area favors the high loading capacity of HRP and GOx. The dual enzymes provide the high catalytic action towards the target analyte and stability of the sensor as well. The sensor showed excellent sensitivity and LOD of 53.89 μA mmol/L.cm^−2^ and 3.99×10^−7^ mol/L, respectively [139].

### 4.6. Detection of Other Markers

Karimi-Maleh and Arotiba reported an electrochemical sensor for simultaneous detection of cholesterol (CL), AA, and UA in serum and urine samples. The sensor was developed by using nanocomposites of copper oxide modified RGO functionalized by IL at carbon paste electrode. The SWV found that the sensor is highly sensitive, and the LOD was 9.0 nM, 9.0 nM, and 0.08 µM for CL, AA, and UA, respectively. In addition, the sensor has high stability, and the detection capacity was reduced to ~93.5% after 25 days. Moreover, the separate oxidation peak was observed for CL, AA, and UA and was able to distinguish from each other. In addition, the proposed sensor exhibits a high sensitivity of 0.0902, 0.0495, and 0.0236 μA/μM for CL, AA, and UA, respectively. The simultaneous detection of three markers through the sensor will be a good candidate for the determination of markers for the prediction of metabolic processes and their responses in the human body [140].

In another context, Jalalvand et al. developed the MIP-based sensor for the determination of cholestenol. Herein, the electrochemical polymerization and deposition of dopamine and Au-Pd bimetallic NPs were performed on the MWCNT-CS-IL electrode surface. Further, the molecular imprinting was done on the modified surface by the electro-polymerization of amino thiophenol as a functional monomer and cholestenol as a template molecule in the ionic solvent in the potential range of -0.5 to 0.8 V for six cycles at 50.0 mV/s scan rate. The impedimetric and voltammetric investigation of cholestenol calculated that the LOD was 0.05 and 0.2 pM, respectively, which are comparatively too close for both techniques. The sensor has excellent reproducibility, sensitivity, and stability for up to seven weeks. Consequently, they observed that the effective imprinting site could be greatly affecting the sensitivity of the sensor. Moreover, the specificity of the sensor is poor, as the concentration of 1.0 µM vitamin D3, 0.1 µM progesterone, and 10.0 µM cholesterol can significantly affect the result [141]. In another study, Boobphahom et al. detected the creatinine in serum sample on a non-enzymatic, electrochemical technique integrated paper-based microfluidic device. They fabricated the paper device by digital wax printing followed by the immobilization of CuO-IL nanocomposites on ERGO. The CuO has a high surface area and electro-catalytic property and tends to form a chelate complex with creatinine. Moreover, IL further improves its electro-catalytic property by enhancing electron transport mobility. Amperometric detection was done to investigate the catalytic action for the copper-creatinine complex, where the linear detection range was calculated to be 0.01-2000 µM and LOD of 0.2 µM. Utilizing a low-cost material for electrode fabrication could turn it into a cost-effective device. Moreover, the one-time usable, disposable nature and biocompatibility attributes make them attractive towards POC on-site diagnostic applications [142]. 

Similarly, RGO functionalized by β-cyclodextrin and (BMIM)(PF_6_) IL was employed for simultaneous detection of guanine and adenine by Wang et al. through the SWV detection technique. β-cyclodextrin (βCD) is a class of oligosaccharides that have a cavity of ~0.66 nm, where the guanine and adenine interact to form the host–guest complex. However, it suffers from poor electro-conductivity. To overcome this issue, it was modified by IL, which improves the electron transport movement and also reduced the GO to RGO as well. Study shows that the concentration of βCD-RGO-IL greatly affects the current response, as observed when concentration of βCD-RGO-IL increased the oxidation current response and at 10.0 µL found maximum response, but further concentration increased to 15.0 µL, resulting in the decrease of oxidation current response for guanine and adenine. This may be attributed to the hindrance of the electron flow owing to the large shape of βCD. The SWV analysis measured the LOD of the sensor for both guanine and adenine at 0.01 µL [143]. Similarly, the detection of guanine and adenine in spiked serum samples through voltammetric sensors was reported by Zhang et al. in 2019. They employed the MnO_2_ NPs-modified GO to construct the electrode. MnO_2_ NPs have a high surface area and are less toxic and are environmentally friendly, but the poor conductivity restricted their application in sensor fabrication. To overcome the poor conductivity issue, they utilized the IL on MnO_2_ NPs-GO, where IL is attached to GO through epoxy ring-opening reaction to enhance the conductivity, and it also reduced the GO to convert RGO, which possesses higher conductivity than GO. Further, a layer of polydopamine (PDA) was coated on a modified electrode surface to construct PDA/MnO_2_/IL-GR/GCE, providing more reaction sites to further improve the sensitivity of the sensor. The voltammetric estimation calculated that the LOD of the sensor was 0.25 and 0.15 µM for guanine and adenine, respectively, and it is also applicable for diagnostics in real samples with satisfactory performance [144].

In another study, Jamei et al. reported an electrochemical aptasensor detection technique for the determination of lysozyme. Since the binding efficiency of the aptasensor is high towards the target analyte, it is a highly specific and sensitive tool. In addition, the high stability and rapid response make it a promising candidate in sensor application. Towards the lysozyme detection, authors fabricated an electrode by amine-functionalized RGO. The functionalization was done through the modification of RGO by (BMIM)Br IL and amino-mesh silica NPs. Further, the anti-lysozyme aptamer was conjugated on the electrode surface using glutaraldehyde cross-linked through the imine complex chemistry. DPV and EIS measurements display that the aptasensor has ultra-low LOD of 2.1 and 4.2 fmol/L, respectively. Further, the sensor could be applicable in a broad range of samples, such as serum tear, urine, egg white, and wine, with reliable performance. A schematic of the step-by-step preparation of the electrochemical sensor is illustrated in Figure 13B [145]. Very recently, Anusha et al. reported an electrochemical sensor for vitamin D_3_ determination in serum and urine. Firstly, they synthesized the Co-Ag/PANI-PPY/IL nanocomposites via the oxidative polymerization process. They mixed the aniline, pyrrole, and (BMIM)(PF_6_) IL in an acidic medium followed by the addition of silver and cobalt salt. However, ammonium persulphate (APS) was added to initiate the polymerization and nanoparticles formation. The aniline, pyrrole, and APS molar ratio was 2:1:3 where the best synthetic results were obtained. SWV analysis was done for the Co-Ag/PANI-PPY/IL/SPE to determine the performance of electrodes and revealed that they have excellent conducting characteristics, which make them highly sensitive sensors. SWV analysis calculated the sensor as having a dynamic detection range from 0.0125 to 22.5 µM with LOD of 0.0073 µM for vitamin D_3_. Furthermore, they extended the work and fabricated a paper-based sensor surface on xerox paper through inkjet printing. The paper-based electrode was analyzed by the chronoamperometric technique, which shows satisfactory performance, and LOD was calculated to be 0.015 µM, which is comparable to nanocomposites-modified SPE electrode, and it was found that the detection ability is considerably good and very close. Such paper-based electrodes could be cost-effective, miniaturized, and easily printed in a less time-consuming fabrication process, which makes them considerable attention in this sensor applications [146].

Ding and Zhang et al. reported a label-free impedimetric sensor based on a carboxyl-functionalized MWCNT and (BMIM)(PF_6_) IL-containing electrode on GCE for detection of insulin-like growth factor I (IGF-I) at picogram of concentration at a very early stage to examine the polycystic ovary syndrome disease. The modified electrode displays high conductivity, but after the immobilization of the IGF-I antibody, the current response decreases. Furthermore, IGF-I antibodies interact with IGF-I antigen in clinical samples to exhibit antigen-antibody chemistry, which further reduced the current response, as measured by the EIS technique. The LOD of the sensor was calculated to be 22.0 pg/mL and has high reproducibility, selectivity, and stability as well [147]. 

## 5. Conclusions and Future Viewpoints

In this review, we summarized the ILs and CNMs hybrid nanocomposites-based electrochemical sensors and biosensors. CNMs, including GR, CNTs, RGO, are conductive and highly stable and have been utilized for electrochemical biosensor fabrication for a long time. On the other hand, ILs have numerous excellent properties, such as high electro-conductivity, surface functionality, wide electrochemical window, good catalytic efficiency, and prevention of the agglomeration of composites used for the surface modification of carbon materials. As a result, the hybrid ILs-CNMs adapt to display superior properties and have better performance than the pure ILs- and CNMs-based electrochemical biosensors. Thus, the sensitivity, stability, detection ranges, and reproducibility of the biosensor could be significantly enhanced. Furthermore, a great deal of research implementation remains for the proper selection of cationic and anionic moieties as well as functionality with the CNMs that could result in promising materials in electrochemical biosensor fabrication, which benefits on-site detection. Moreover, the application of ILs-CNMs needs to be explored and should be extended to not only the sensors and biosensors but also for the betterment of other devices, such as a super-capacitor, batteries, solar cells, etc.

## Figures and Tables

**Table 1:** Conductivity and electrochemical stability of ILs. (mS/cm- milliSiemens/centimeter; V-volt; cP- centipoise).

Ionic Liquids	Conductivity (in mS/cm)	Electrochemical Window (in V)	Viscosity (in cP)	Ref.
[BMIM][BF_4_]	3.5	-	154	[88]
[BMIM][NTf_2_]	4.0	-	52	
[BMIM][PF_6_]	1.46–1.0	-	308	
[BMIM] [OTf]	2.9	-	90	
[EMIM] [NTf_2_]	9.1	-	28–34	
[EMIM][PF_6_]	5.2	-	-	
[EMIM][OTf]	8.6		45	
[EMIM][BF_4_]	12.0	4.3	33.8	[89]
[EMIM][F_3_MeS]	8.6	4.3	39.8	
[EMIM][SCN]	27.0	2.9	24.7	
[EMIM][DCA]	21.0	2.3	14.6	
[Pyr14][Tf_2_N]	2.1	6.6	0.002	
[N8,8,8,1][NTf_2_]	2.2	5.7	530	
[Et_3_S][NTf_2_]	8.2	5.5	5.2	

**Table 2:** Ionic liquids-carbon hybrid nanoparticles-based common electrochemical sensor and biosensor for detection of relevant molecules.

Nanomaterial	Ionic Liquid	Target Analyte	Sample	Technique	Linearity	LOD	Ref.
Cancer biomarker							
GQDs-ILs-nafion	[BDMIM][BF_4_]	CEA	Serum	DPV	0.5 fg/mL–0.5 ng/mL	0.34 fg/mL	[112]
AuNPs/IL/PICA/RGO/GCE	[BMIM][PF_6_]	CEA	Spiked serum samples	DPV	0.02–90.0 ng/mL	0.02 ng/mL	[113]
IL/RGO/AuNPs	[APMIM]Cl	CEA, AFP	Serum	DPV	0.01–100 ng/mL	CEA 0.01 ng/mL, AFP 0.006 ng/mL	[114]
GR-IL-Pt and Ti_3_C_2_ MXenes-AuNPs hybrids	[BMIM][PF_6_]	CEA	Serum	ECL	0.1 pg/mL–10.0 ng/mL	34.58 fg/mL	[115]
GR-IL-pPT	[BMIM][PF_6_]	CEA	Serum	ECL	0.001 fg/mL–1.0 ng/mL	0.0003 fg/mL	[116]
Au-CoS/GR and CeO_2_/IL doped with carboxymethyl chitosan	[BP][BF_4_]	PSA	Serum	DPV	0.5 pg/mL–50.0 ng/mL	0.16 pg/mL	[117]
3D porous cryogel (CS-GR-IL-Fc cry)	[BMIM][TFSI]	PSA	Serum	DPV	1.0 × 10^−7^–1.0 × 10^−1^ ng/mL	4.8 × 10^−8^ Ng/mL	[118]
IL-CA-PGEs	[BMIM][PF_6_]	miRNA-34a	FBS	EIS	2.0–10.0 µg/mL	0.826 µg/mL	[119]
IL-CA-PGEs	[BMIM][PF_6_]	miRNA-34a	FBS	DPV	1.0–7.0 µg/mL	0.40 µg/mL	[120]
NH_2_-IL-RGO	3-(2-aminoethyl)-1-propyl-1H-imidazol-3-ium chloride	HPV16 DNA	-	DPV	8.5 nM–10.7 µM	1.3 nM	[121]
Cardiovascular biomarker							
CIL-HCNTs and IgG Ab-Fc-COOH	[CMMIM]Cl	cTnI	Serum	DPV	0.01–60.0 ng/mL	0.006 ng/mL	[122]
Dialdehyde-functionalized Ionic Liquid and Helical Carbon Nanotubes (DIL−HCNT)	4-(Bromomethyl) benzaldehyde, 4,4′-Bipyridine	cTnI	-	DPV	0.05–30.0 ng/mL	0.03 ng/mL	[123]
Immunoglobulins							
CNT-IL	Aldehyde terminated IL	IgG	Serum	DPV	0.1–15.0 ng/mL	0.02 ng/mL	[124]
CNT/Fc-IL-CHO	-	IgG	-	DPV	0.05–30.0 ng/mL	0.01 ng/mL	[125]
Nf/Fc-IL-CHO	-	IgG	-	DPV	0.05–35.0 ng/mL	0.03 ng/mL	[126]
ZnO/MPC/IL	-	CRP	-	DPV	0.01–1000 ng/mL	5.0 pg/mL	[127]
MIPs/CS/IL-GR	[BMIM][PF_6_]	BSA	Plasma	DPV	1.0 × 10^−10^–1.0 × 10^−4^ g/L	2 × 10^−11^ g/L	[128]
Neurotransmitter							
GO/IL/AuNPs	1-butyl-3-methyl imidazole hydrobromide	DA	Urine	DPV	7.0 nM–5.0 mM	2.3 nM	[129]
C-dots/IL-GR	1-methylimidazole and 2-bromoethylamine hydrobromide	DA	Spiked fetal bovine serum	DPV	0.1–600 μM	30.0 nM	[130]
GQDs/IL-SPCE	[BMIM][PF_6_]	AA, DA, UA	-	DPV	25.0–400 μM, 0.2–10.0 μM, and 0.5–20.0 μM,	6.64, 0.06 and 0.03 μM,	[131]
Cs-SWCNT/IL	[BMIM][BF_4_]	DA, UA	Urine	LSV	DA 0.50–30.0 μmol/L, UA 0.50–1000 μmol/L	DA 0.16 μmol/L, UA 0.17 μmol/L	[132]
AChE-ChO/GO/IL	[AMIM][TFSI]	Ac, ACh	Serum	ADPSV	5.0–1000 nmol/L	0.885 nmol/L, 1.352 nmol/L	[133]
Glucose							
IL/RGO/Ni-Pd	1,2-Dimethylimidazole, 1-bromobutan	Glucose	Serum	Amperometric	0.2–10.0 mM	0.03 μM	[134]
Co-salophen-IL/ERGO/SPE	-	Glucose	Serum and urine	Amperometric	0.2 μM–1.8 mM	0.79 μM	[135]
CHO/IL/ERGO Azu-A/CHO-IL/SPE	(3-(3-formyl-4-hydroxy benzyl)-3- methylimidazolium hexafluorophosphate	H_2_O_2_, Glucose	H_2_O_2_ in milk, Juice, glucose in human serum	Amperometric, CV	0.03–1.0 mM 0.05–2.4 mM	11.5 and 17.0 μM	[136]
CuO-IL/RGO	-	Glucose	Urine	Chronoamperometry	0.03–7.0 mM	0.14 μM	[137]
CoPc/G/IL/SPCE	[BDMIM][BF_4_]	Glucose	Food, Serum	Chronoamperograms	0.01–1.3 mM and 1.3–5.0 mM	0.67 μM	[138]
IL-GR-CNTs	1-Methylimidazole	Glucose	-	DPV	0.004–5.0 mmol/L	3.99 × 10^−7^ mol/L	[139]
Other markers							
CuO-rGR/1M3OIDTFB	[OMIM][BF_4_]	CA, AA, UA	Real samples	SWV	0.04–300 μM, 0.04–240 μM, 0.4–400 μM	9.0, 9.0, and 0.08 μM	[140]
MIP/AuPd NPs/PDA/MWCNTs-CS-IL/GCE	[EMIM][BTI]	Cholestanol	-	EIS, DPV	EIS: 0.1–60.0 pM and DPV: 1.0–50 pM	EIS: 0.05 pM, DPV: 0.2 pM	[141]
CuO/IL/ERGO	[BDMIM][BF_4_]	Creatinine	Serum	Paper-based microfluidic	0.01–2.0 mM	0.22 mM	[142]
βCD/RGO/IL	[BMIM][PF_6_]	Guanine, Adenine	-	SWV	Guanine: 0.03–10.0 mM and adenine: 0.02–7.0 mM	0.01 mM	[143]
PDA/MnO_2_/IL-GR	2-bromoethylamine hydrobromide, 1-methyl imidazole,	Guanine, Adenine	Blood	DPV	10.0–300 μM	Guanine: 0.25 μM and Adenine: 0.15 μM	[144]
Amino-rGO/IL/Amino-MSNs	[BMIM]Br	Lysozyme	-	EIS, DPV	EIS: 10.0 fmol/L–200.0 nmol/L and DPV: 20.0 fmol/L–50.0 nmol/L	2.1 and 4.2 fmol/L	[145]
Co-Ag/PANI-PPY/IL@GCE	[BMIM][PF_6_]	Vitamin D3	Serum and urine	SWV	0.0125–22.5 μM and 0.025–0.125 μM	0.0073 and 0.015 μM	[146]
MWCNT/IL/GCE	[BMIM][PF_6_]	IGF-1		EIS	0.4–15.0 ng/mL	22.0 pg/mL	[147]

## Abbreviations

List of abbreviations of commonly used ionic liquids and others.

**Abbreviation**	**Name**
[BMIM][BF_4_]	1-Butyl-3-Methylimidazolium Tetrafluoroborate
[EMIM][BF_4_]	1-Ethyl-3-Methylimidazolium Tetrafluoroborate
[AMIM][TFSI]	1-Allyl-3-Methylimidazolium Bis(trifluoromethylsulfonyl)imide
[BMIM][TFSI]	1-Butyl-3-Methyllimidazolium Bis(tri-fluoromethylsulfonyl) imide
[APMIM]Cl	1-Aminopropyl-3-Methylimidazolium Chloride
[BMIM]Cl	1-Butyl-3-Methylimidazolium Chloride
[BMIM]Br	1-Butyl-3-Methylimidazolium Bromide
[DMIM]Br	1-Decyl-3-Methylimidazolium Bromide
[BMIM][PF_6_]	1-Butyl-3-Methyliidazolium Hexafluorophosphate
[BMIM][TFSI]	1-butyl-3-methyllimidazoliumbis (tri-fluoromethylsulfonyl) imide
[BP][BF_4_]	1-Butyl-Pyridine Tetrafluoroborate
[OMIM][BF_4_]	1-Methyl-3-Octylimidazolium Tetrafluoroborate
[EMIM][BTI]	1-Ethyl-3-Methylimidazolium Bis(tri-fluoromethylsulfonyl)imide
[BMIM][BTI]	1-Butyl-3-Methyllimidazolium Bis(tri-fluoromethylsulfonyl)imide
[BDMIM][BF_4_]	1-Butyl-2,3-Dimethylimidazolium Tetrafluoroborate
[BMIM][Ac]	1-Butyl-3-Methylimidazolium Acetate
[BMIM][NTf_2_]	1-Butyl-3-Methyl Imidazolium Bis(trifluoro-methylsulfonyl)amide
[VEIM][BF_4_]	1-Vinyl-3-Ethylimidazolium Tetrafluoroborate
[NMIM][Cl]	1-(nonyl)-3-methylimidazolium chloride
[CMMIM]Cl	Carboxymethyl-3-Methylimidazolium Chloride
[EMIM][F_3_MeS]	1-Ethyl-3-Methylimidazolium Trifluoromethyl Sulfonate
[EMIM][SCN]	1-Ethyl-3-Methylimidazolium Thiocyanate
[EMIM][DCA]	1-Ethyl-3-Methylimidazolium Dicyanamide
[Pyr14][Tf_2_N]	N-Butyl-N-Methylpyrrolidinium Bis(trifluoromethylsulfonyl)imide
[N8,8,8,1][NTf_2_]	N-Methyl-N-Trioctylammonium Bis(trifluoromethylsulfonyl)imide
[Et_3_S][NTf_2_]	Triethylsulphonium Bis(trifluoromethylsulfonyl)imide
